# Resting-state EEG and MEG gamma frequencies in schizophrenia: a systematic review and exploratory power-spectrum meta-analysis

**DOI:** 10.1038/s41537-025-00596-z

**Published:** 2025-03-21

**Authors:** Marco De Pieri, Michel Sabe, Vincent Rochas, Greta Poglia, Javier Bartolomei, Matthias Kirschner, Stefan Kaiser

**Affiliations:** 1 Division of Adult Psychiatry, Department of Psychiatry, University Hospitals of Geneva, Thonex, Switzerland; 2 Faculty of Medicine, University of Geneva, Geneva, Switzerland; 3 Functional Brain Mapping Laboratory, Department of Basic Neurosciences, University of Geneva, Geneva, Switzerland

**Keywords:** Biomarkers, Neural circuits

## Abstract

The hypoactivity of parvalbumin-containing interneurons (PV-interneurons) is a pathogenetic mechanism of schizophrenia according to the glutamatergic theory, and PV-interneurons are necessary for the generation of EEG/MEG gamma-frequencies (30–100 Hz). The present study aims to a literature synthesis on resting-state gamma-frequency changes in patients with schizophrenia vs healthy controls, and to examine the relationship between these changes and severity of symptoms. A protocol was enregistered in PROSPERO and a systematic search was conducted in PubMed, PsycINFO and Cochrane Database of Systematic Reviews, following PRISMA guidelines. An exploratory meta-analysis was realized. Out of 1391 records, 43 were included for a qualitative synthesis (*N* = 2133 [11–185], females 37.4%, age 33.9 ± 9.2). Results on power spectra were heterogeneous: in 12 studies gamma power was increased, involving the whole brain (*N* = 3), multiple regions (*N* = 6) or only frontal (*N* = 1), central (*n* = 1) and temporal (*N* = 1) areas; in 3 studies gamma power was reduced, involving multiple areas (*N* = 2) or the right temporal region (*N* = 1); one study revealed mixed results and 13 studies showed no differences. The meta-analysis on 4 studies (*N* = 211) showed non-significant differences between patients and controls and a large heterogeneity. The functional connectivity picture consists of sparse patterns of decreases and/or increases, widespread to multiple regions. Relationships emerged between gamma power and connectivity and severity of psychotic and cognitive symptoms. Theta-gamma coupling was increased in patients, with limited evidence for other changes in phase-amplitude coupling. Resting-state gamma-frequencies alterations in schizophrenia were inconsistent across studies; the heterogeneity of patients and methods could partially explain this outcome.

## Introduction

Schizophrenia is a complex disorder consisting of several dimensions: positive symptoms (i.e., delusions and hallucinations), cognitive dysfunction (involving executive functions, memory, complex attention, language and social cognition), amotivation (including anhedonia, avolition and asociality) and diminished expression (including blunted affects and alogia)^1^. With a lifetime prevalence of 0.7–1%, the personal and societal burden of the disease is high^2^.

The glutamatergic theory of schizophrenia proposes the hypoactivity of the N-methyl-D-aspartate (NMDA) glutamate receptors located on the gamma amino-butyric acid (GABA) parvalbumin-containing interneurons (PV-interneurons) as a core pathogenetic process. NMDA receptor hypofunction is sensed by PV-interneurons as a lack of pyramidal neurons activity, leading to a decrease in inhibitory tone^3^. The consequent excitation/inhibition imbalance leads to the hyperactivation of cortical pyramidal neurons^4,5^, which in turn also disrupts the activities of the dopaminergic mesolimbic and mesocortical systems^6,7^.

Electroencephalography (EEG) and magnetoencephalography (MEG) are neuroimaging techniques, with high temporal and spatial resolutions, which detect brain activity as an ensemble of electro-magnetic oscillations^8^. MEG allows for a better spatial resolution, because the magnetic signal is not distorted when passing through tissues with different conductivities, differently from EEG, and is also not prone to contaminations from muscle and cardiac artifacts. However, MEG cannot detect sources located radially to the sensor; thus, EEG and MEG can be considered as complementary for an accurate signal modeling^9^.

EEG/MEG gamma frequencies are brain oscillations in the 30–100 hz range^9,10^, originating from a complex interplay between pyramidal neurons and PV-interneurons, and are linked to the abovementioned glutamatergic theory of schizophrenia. In fact, according to the pyramidal-interneuron network gamma model (PING), gamma activity is generated through feedback inhibition on pyramidal neurons by PV-interneurons^10,11^ occurring in all regions^10,11^; the decay rate of this inhibition, approximately 25 ms, particularly contributes to the generation of 40 Hz oscillations^12,13^. The PING model has mostly replaced other models explaining the origin of gamma frequencies: the interneuron gamma model (ING), which posits a simultaneous firing of pyramidal cells and interneurons^14^, and the I-I model, based on a network of only two inhibitor interneurons connected^11^.

Gamma frequencies could represent an in vivo marker of the pathophysiological process underlying schizophrenia, and are easily accessible via EEG/MEG, even if it remains uncertain whether this reflects the primary pathophysiological mechanism or a downstream phenomenon. The relevance of gamma oscillations is corroborated by their physiological increase during adolescence, the typical age of onset of schizophrenia^15^, and by their abnormality already at illness onset^16–18^.

Gamma frequencies have been associated with sensation, perception, attention, memory, cognitive processing, consciousness and to synaptic plasticity, indicating their key role in brain functioning, and their possible implication in whole-brain diseases, such as schizophrenia^3,16,19–21^. Neuroimaging studies have suggested that treatment-resistant schizophrenia may rely more on a glutamatergic than on a dopaminergic dysfunction^22^.

Several studies have investigated the modification of gamma frequencies in patients with schizophrenia and their relationship with perceptual, affective and cognitive functioning. The resting state was the most common experimental setting, but a synthesis of evidence is missing; fewer studies evaluated task-related EEG, and they have already been summarized in important systematic reviews^23–25^.

Resting-state gamma frequencies and their relationship with the NMDA receptor functioning were analyzed in preclinical settings, as summarized by a recent review and perspective paper by Bianciardi and Uhlhaas. Their review indicates that in both animal models and healthy human subjects the pharmacological antagonism to the NMDA receptor results in an increase in resting-state gamma-band activity^26^, which was also related to psychotic symptoms in one study^27^. Moreover, a genetically determined reduction in the NMDA receptor activity, provoked an increased gamma power^28–31^ which was correlated with schizophrenia-like behaviors in animal models^32–34^, and with schizophrenia in humans^35^.

Following these lines of evidence, an increased resting-state gamma power in patients with schizophrenia compared to healthy subjects would be expected. However, the review of Uhlhaas and Bianciardi revealed a more complex picture, with resting-state studies on patient with schizophrenia showing an increase or a decrease of power and functional connectivity across brain areas^26^. With the present paper, we aimed to update the work of Bianciardi and Uhlhaas on patients with schizophrenia, concerning the following three aspects: (i) inclusion of task related studies involving a resting-state phase; (ii) association of gamma-band alteration with symptoms’ severity; (iii) inclusion of studies on phase amplitude coupling and on functional connectivity measures not considered before.

In fact, the objective of the present systematic review and exploratory meta-analysis was to evaluate resting-state gamma-band related features in patients with schizophrenia, including differences from a comparator group (i.e., controls subjects, first-degree relatives or patients with another mental disorder), and their relationship with disease duration and severity, in several psychopathology domains.

## Methods

### Study design

The Preferred Reporting Items for Systematic Reviews (PRISMA) statement was followed to design and conduct the systematic review. We performed a comprehensive literature search on EEG and MEG resting-state studies evaluating the gamma frequency range.

A review protocol was enregistered in PROSPERO (CRD 42024511291).

### Article search strategy

A systematic literature search was conducted in three electronic databases: PubMed, PsycINFO and the Cochrane Database of Systematic Reviews since inception to 30th of September 2024 with no time limit and with the English language as the only selected filter. Moreover, a snowball search (i.e., a research method where an initial source, such as a relevant article or study, is used to find additional sources by examining its references or citing articles) was performed.

The following combination of search terms was used:

*(“EEG” OR “electroencephalography” OR “electroencephalogram” OR “MEG” OR “magnetoencephalography”) AND “resting-state” AND (“schizophrenia” OR “first episode of psychosis”).*

### Selection process and criteria

First, any duplicate data from the combination of the three databases were excluded. The remaining articles were included in the systematic review only if they met the following criteria:

*Inclusion criteria*

1. Clinical trials, case-control and cohort studies;
2. studies carried out in humans;
3. studies published in the English language;
4. studies conducted on patients affected by a disorder included in the DSM5 chapter “schizophrenia spectrum disorder”
5. studies including subjects aged between 18 and 65 years

*Exclusion criteria*

1. books chapters, comments, editorials, case reports, theses, proceedings, letters, short surveys, notes;
2. studies focused on EEG changes induced by a treatment (e.g., medications, psychotherapy, or meditative practices);
3. studies on sleep EEG;
4. studies in which first-episode of psychosis patients did not transition to schizophrenia, or focused on individuals at clinical high-risk for psychosis;
5. studies irrelevant to the topic.

Two researchers (MDP and MS) independently screened for the eligibility all of the articles based on titles and abstracts and then proceeded to read the full text. Any disagreements were resolved by consensus or by the decision of a third reviewers.

### Data extraction

MDP and MS recorded the following variables from each included article: author(s), year of publication, sample size, study design, sociodemographic and clinical features, assessment instruments for diagnosis, current and past pharmacological treatments, EEG methods and results, and statistical calculations.

The Assessing the methodological quality of systematic reviews (AMSTAR 2 checklist)^36^ and the Newcastle-Ottawa scale (NOS) for assessing the quality of non-randomized case control studies^37^ were used to assess the quality and completeness of the data. Some of the NOS items were rule out and scoring was adapted, given that they were not applicable to the observational, cross-sectional studies reviewed here.

Data files and the R script for the meta-analysis can be found on the open science framework (OSF).

### Summary measures

Statistical analyses were conducted in RStudio (RStudio team, Version: 2023.12.1 + 402) using R (R Core Team, 2024, version 4.3.3). We used the esc package to compute the effect sizes and the meta, metaphor and dmetar packages to conduct the meta-analyses.

Hedge’s g statistics were calculated for the standardized mean difference in bandwidth power between patients and control subjects; Hedge’s g was preferred to Cohen’s d in order to adjust for the small sample size bias^38^. Given that all effect sizes should be independent, we conducted 4 separate meta-analyses on the four main brain regions. We focused on the gamma1 band (i.e., 30–50 Hz), which has been more consistently reported across studies, while data were insufficient to conduct meta-analyses in the gamma2 and gamma3 subbands.

### Synthesis of results

Given the high methodological heterogeneity among studies, a random effect model was used to combine and weight effect sizes across studies, using inverse variance methods. Heterogeneity was quantified using the percentage of variability in the effect size (i.e., the *I*^2^ statistic). A value ≥ 75% indicates high heterogeneity, a value of 50% indicates moderate heterogeneity, a value of 25% indicates a low heterogeneity^39^. Additionally, the between-study variance estimator τ^2^ using the Hartung-Knapp-Sidik-Jonkman method was calculated, and heterogeneity was moreover statistically assessed with the Cochran’s *Q* statistic. An influence analysis was carried out by visual inspection of Baujat^40^ and Viechtbauer-Cheung^41^ graphs.

## Results

### Characteristics of the included studies

**Fig. 1:**
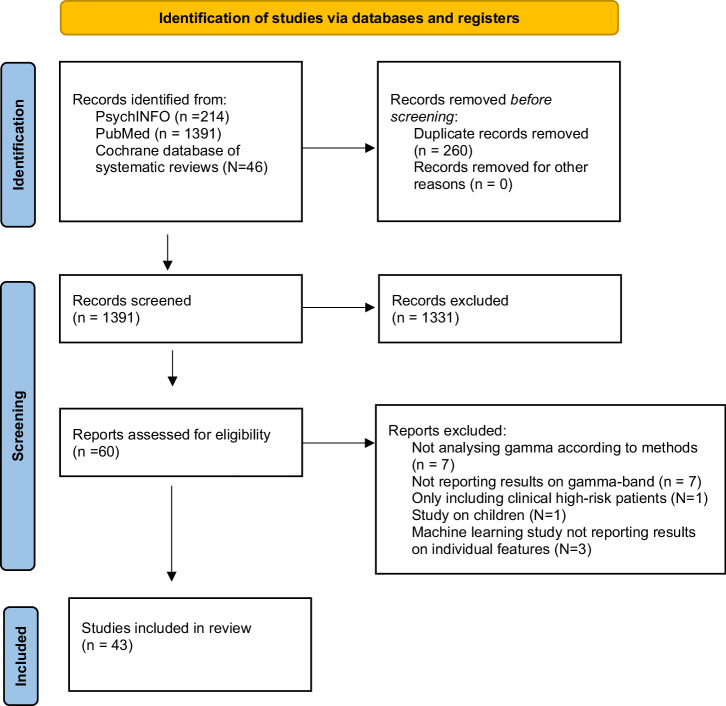
PRISMA 2020 flowchart of study selection.

Figure 1 shows the selection process. The combined outcome of the three databases yielded a total of 1651 records, and no studies were added by hand search. Of the total studies, 260 were duplicates, leaving 1391 articles. After reading the titles and abstracts, 1331 articles were excluded because they were not relevant to the topic or did not meet the inclusion and exclusion criteria. The full texts of 60 articles were examined in detail. Six studies were excluded because they focused on EEG-bands other than gamma, 7 were excluded because the results did not report any positive or negative results on gamma-related features, 1 was excluded because the study was conducted on clinical high risk for psychosis patients only, and 1 was excluded because the study reported on children. Three studies using machine learning methods were excluded because they did not report the link between each feature under study and the variable of interest. No studies were excluded due to full-text unavailability or unavailability of English full-text.

A total number of 43 studies were ultimately identified as eligible for inclusion in the current review.

Tables 1–3 summarize the characteristics of the studies, including sociodemographic, clinical and EEG-related features.

**Table 1:** Sociodemographic features.

Reference	Diagnosis	Status	*N*	Age (years)	Age of onset	Sex (F)	Duration of illness (months)	Education (years)	Handedness (Right)
***Rutter 2009***	SZ	Na	38	31.2 ± 9.8	Na	11	Na	Na	Na
***Venables 2009***	SZ	Stabilized outpatients	48	44.8 ± 9.6	Na	11	Na	13.9 ± 2.6	Na
***Kikuchi 2011***	SZ	Outpatients	21	28.1 (18–48)	Na	10	24.2 (1–140)	Na	Na
***Hanslmayr 2012***	SZ	Inpatients	26	27.35 ± 5.66	Na	5	Na	Grade 3	25
***Andreou 2014a***	FES	Na	22	24.09 ± 5.1	Na	3	14.16 ± 15.6	Na	Na
***Andreou 2014b***	FES	Outpatients and stabilized inpatients	19	23.53 ± 4.3	Na	2	Na	13.66 ± 3.3	Na
***Garakh 2014***	FES, SA	Inpatients	32	28.91 ± 10.64	28.78 ± 10.54	16	Na	13.47 ± 1.50	32
***Kam 2014***	SZ	Outpatients	132	40	Na	40	Na	High school completed	Na
***Kim 2014***	SZ	Outpatients	20	22.80 ± 3.91	Na	4	70 ± 36	12.30 ± 1.83	18
***Di Lorenzo 2015***	SZ	Outpatients	77	35.44 ± 11.05	21.13 ± 4.82	26	171.7 ± 110	12.75 ± 3.32	Na
***Hirano 2015***	SZ	Na	18	45.4 ± 8.9	Na	3	234 ± 114	13.5 ± 2.1	15
***Mitra 2015***	SZ	Na	15	28.87 ± 6.81	Na	3	55.13	Grade 6 or less (13.3%); grade 7 to 12 without graduating (46.7%); High school graduate (3 6.7%); College graduate (33.3%)	15
***Tikka 2015***	SZ	Na	30	29.97 ± 8.29	Na	10	63.2 ± 6.31	Illiterate (*N* = 5); Primary ( = 7); Secondary (*N* = 15); Graduate (*N* = 3)	30
***Ramyead 2016***	FES	Na	31	30.8 ± 8.92	Na	13	Na	10.5 ± 2.99	Na
***Umesh 2016***	SZ	Outpatients	20	29.8 ± 7.68	23.47 ± 7.35	0	Na	12.4 ± 1.84	20
***Won 2017***	SZ	Na	90	37.44 ± 10.25	26.78 ± 8.94	49	121.6 ± 95	Na	Na
***Arikan 2018***	SZ	Outpatients	23	39	Na	Na	>6	11.5	Na
***Baradits 2018***	SZ	Na	60	35.2 ± 9.6	Na	29	120 ± 102.8	College degree: Yes (*N* = 14), No (*N* = 46)	Na
***Grent’t’Jong 2018***	FES	Na	21	27.0 ± 1.5	Na	5	Na	14.1 ± 0.7	Na
	Chronic SZ		34	37.1 ± 2.0	Na	12	Na	14.2 ± 0.6	
***Hirano 2018***	SZ	Na	18	45.4 ± 8.9	Na	3	234 ± 114	13.5 ± 2.1	13
***Jonak 2018***	FES	Na	30	22.31 ± 3.11	Na	15	20.11 ± 16.51	13.87 ± 1.88	Na
	chronic SZ	Na	30	36.71 ± 9.05	Na	18	132.21 ± 92.83	13.63 ± 3.24	
***Krukow 2018***	SZ	Stabilized inpatients	32	21.30 ± 3.10	Na	17	12.66 ± 6.33	13.80 ± 1.88	Na
***Takahashi 2018***	SZ	Outpatients	21	28.1 ± 10.1	Na	10	24.2 ± 36.2	14.1 ± 1.9	21
***Zeev-Wolf 2018***	SZ	Inpatients and outpatients	39	38.74 ± 9.91	Na	38	Na	11.38 ± 2.86	41
***Lottman 2019***	FES	Inpatients and outpatients	21	23.52 ± 4.64	Na	6	18.58 ± 26.68	12.85 ± 0.59	Na
***Vignapiano 2019***	SZ	Outpatients	145	36.94 ± 9.49	Na	47	158.52 ± 100.44	12.23 ± 3.05	Na
***Alamian 2020***	SZ	Na	25	44.96 ± 8.55	Na	8	Na	Na	Na
***Freche 2020***	SZ	Inpatients	21	40 ± 8	Na	2	232 ± 96	Na	Na
***Kim 2020***	SZ	Na	38	43.16 ± 11.16	30.06 ± 10.94	22	145 ± 90.6	12.92 ± 273	Na
***Krukow 2020***	FES	Outpatients	34	21.15 ± 2.95	Na	18	12.68 ± 6.31	13.76 ± 1.82	34
***Lee 2020***	FES	Inpatients and outpatients	59	23.2 ± 4.9	Na	37	Na	13.8 ± 2.2	54
***Soni 2020***	SZ	Outpatients	32	27.31 ± 6.24	Na	10	Na	Na	32
***Tanaka-Koshiyama 2020***	SZ	Na	157	46.4 ± 10.8	Na	54	300 ± 144	12.4 ± 2.1	Na
***Koshiyama 2021a***	SZ	Outpatients	148	46.2 ± 10.9	Na	51	305 ± 141.6	Na	Na
***Koshiyama 2021b***	SZ	Outpatients	142	44.8 ± 10	Na	53	283.2 ± 133.2	12.4 ± 2.1	Na
***Sun 2021***	SZ	Outpatients	30	29.07 ± 5.67	Na	9	8.23 ± 7.93	13.47 ± 2.29	30
***Yadav 2021***	SSD	Na	29	25.83 ± 4.64	Na	0	13.28 ± 6.75	Primary (*N* = 14); Secondary (*N* = 10); Graduation (*N* = 2); Professional (*N* = 1)	29
***Gordillo 2022***	SZ	Inpatients and outpatients	121	35.8 ± 9.2	Na	22	129.6 ± 104.4	13.3 ± 2.6	115
***Tagawa 2022***	SZ	Na	29	40.8 ± 6.7	Na	17	Na	Na	29
***Ibanez-Molina 2023***	SZ, SA	Na	11	36.23 ± 10.28	Na	2	188.64 ± 120.5	Primary (*N* = 2); Secondary (*N* = 8); Tertiary (*N* = 1)	11
***Jacob 2023***	SZ	Outpatients	57	35.6 ± 13.9	Na	46	Na	Na	52
***Yeh 2023***	SZ	Outpatients	72	42.90 ± 10.87	Na	33	209.3 ± 132	13.31 ± 3.02	Na
***Chang 2024***	SZ	Inpatients	15	40.40 ± 8.68	Na	7	Na	10.53 ± 2.50	Na

Data expressed as mean ± SD. *Na* not available, *FES* first episode of schizophrenia, *SA* schizoaffective disorder, *SSD* schizophrenia spectrum disorders, *SZ* schizophrenia.

**Table 2:** Severity of disease, treatment characteristics.

Reference		Rating scale	Score	Medication status	Antipsychotic	Dose	Other medications
***Rutter 2009***		*Na*	/	Na	Na	Na	Na
***Venables 2009***		*BPRS*	41.8 ± 11.4	Antipsychotic monotherapy	Na	747.7 ± 606 chlorpromazine equivalents	Antiparkinsonian (*N* = 12), Antidepressant (*N* = 25), Mood stabilizer (*N* = 6)
***Kikuchi 2011***		*BPRS*	52.6 (28–76)	Drug naïve	/	/	/
***Hanslmayr 2012***		*PANSS*	Na	Antipsychotic mono- (*N* = 22) or polytherapy (*N* = 1), drug-free (*N* = 3)	FGA and SGA	466 chlorpromazine equivalents	SSRI or tetracyclic antidepressants (*N* = 4)
***Andreou 2014a***		*PANSS*	56 ± 15.6	Antipsychotic monotherapy (*N* = 18) Drug free (*N* = 4)	Amisulpride (*N* = 1), Aripiprazole (*N* = 3), Olanzapine (*N* = 5), Paliperidone (*N* = 3), Quetiapine (*N* = 2), Risperidone (*N* = 5)	188.64 ± 181 chlorpromazine equivalents	Antidepressant (*N* = 6)
***Andreou 2014b***		*PANSS*	54.88 ± 16.5	Antipsychotic monotherapy (*N* = 16), Drug-free (*N* = 3)	Amisulpride (*N* = 1), Aripiprazole (*N* = 2), Olanzapi ne (*N* = 4), Paliperidone (*N* = 3), Quetiapine (*N* = 2), Risperidone (*N* = 4)	239.59 ± 182.4 chlorpromazine equivalents	Antidepressant (*N* = 6)
		*VLMT learning*	59 ± 9.5				
		*VLMT delayed recall*	10.53 ± 3.0				
		*WLM immediate recall*	23.47 ± 7.4				
		*WLM delayed recall*	19.35 ± 9.9				
		*TMT-A*	24.18 ± 7”				
		*TMT-B*	60.41 ± 21.7”				
		*Digit span forward*	7.47 ± 1.9				
		*Digit span backward*	5.93 ± 1.7				
		*Letter-number sequencing*	15.13 ± 2.9				
		*Digit symbol*	51.82 ± 8.6				
		*Letter fluency*	38.56 ± 11.5				
***Garakh 2014***		*PANSS*		Antipsychotic mono- or polytherapy (*N* = 32), Drug naïve (*N* = 10)	Na	227.55 ± 69.10 chlorpromazine equivalents	Mood stabilizers, antidepressants (*N* = na)
		*Positive*	19.34 ± 5.58				
		*Negative*	17.09 ± 4.62				
		*General*	41.50 ± 7.55				
***Kam 2014***		*PANSS*	62.1 ± 16.7	Atypical antipsychotic (*N* = 95) Typical antipsychotic (*N* = 25) Drug free (*N* = 20)	Na	Na	Antidepressants (*N* = 46), Antiepileptics (*N* = 31), Anticholinergics (*N* = 24), Benzodiazepines (*N* = 21), Lithium (*N* = 5), Antiparkinsonian (*N* = 4)
***Kim 2014***		*PANSS*	54.20 ± 12.89	Antipsychotic monotherapy	SGA	Na	Na
***Di Lorenzo 2015***		*PANSS*	76.68 ± 6.29	Antipsychotic mono- (*N* = 62) or polytherapy (*N* = 15)	SGA (*N* = 65), FGA (*n* = 21)	306.10 ± 167.3 chlorpromazine equivalents	Antidepressant (*N* = 18) Benzodiazepine (*N* = 18), Antiepileptic (*N* = 10), Anticholinergic (*N* = 7)
		*SOFAS*	55.61 ± 15.85				
***Hirano 2015***		*SAPS*	9.33 ± 4.90	Antipsychotic mono- (*N* = 13) or polytherapy (*N* = 3) Drug-free (*N* = 2)	SGA (*N* = 16), FGA (*N* = 6)	408.27 ± 454.26 chlorpromazine equivalents	Na
		*SANS*	9.38 ± 5.64				
***Mitra 2015***		*PANSS*	83.87 ± 15.9	Antipsychotic monotherapy	Olanzapine, risperidone, haloperidol	516.62 ± 208.6 chlorpromazine equivalents	
***Tikka 2015***		*PANSS*	82.7 ± 13.73	Drug free or drug naive	/	/	No
***Ramyaed 2016***		*BPRS*	55.0 ± 12.1	Antipsychotic naïve	/	/	Antidepressant (*N* = 4) Benzodiazepines (*N* = 8)
***Won 2017***		*Na*	/	Drug naïve or drug free ( > 1 month)	/	/	No
***Arikan 2018***		*Schedule for assessing the three components of insight*	Na	Drug free (3 wks)	/	/	No
		*SANS*	Na				
		*SAPS*	Na				
***Baradits 2018***		*PANSS*	87.6 ± 17.91	Antipsychotic mono- or polytherapy	Na	599 ± 333.47 chlorpromazine equivalents	Benzodiazepines (*N* = 38)
		*BPRS*	30.76 ± 6.43				
***Grent’t’Jong 2018***	FES	*PANSS*	66.9 ± 3.2	Drug-naïve	/	/	Na
		*BACS (composite score)*	−0.22 ± 0.35				
		*CAARMS total*	25.0 ± 2.4				
	Chronic SZ	*PANSS*	56.3 ± 3.0	Antipsychotic mono- or polytherapy	Na	Na	Na
		*BACS (composite score)*	−1.03 ± 0.21				
		*CAARMS total*	37.6 ± 2.8				
***Hirano 2018***	SZ	*SAPS positive symptoms total*	10.06 ± 5.8	Antipsychotic mono- or polytherapy	SGA (*n* = 12), FGA (*N* = 1), FGA + SGA (*N* = 3), Drug-free (*N* = 2)	408 ± 454 chlorpromazine equivalents	Na
		*SANS negative symptoms total*	8.77 ± 4.5				
***Jonak 2018***	FES	*PANSS*	68.36 ± 11.89	Antipsychotic mono- or polytherapy	Na	346.69 ± 73.91 chlorpromazine equivalents	Na
	Chronic SZ	*PANSS*	72.41 ± 11.02			537.67 ± 203.08 chlorpromazine equivalents	
***Krukow 2018***	SZ	*PANSS*	67.33 ± 12.33	Antipsychotic monotherapy	Olanzapine 56.25% Quetiapine 18.75% Risperidone 15.65% Aripiprazole 9.37%	4.93 ± 1.47 risperidone equivalents	Antidepressants (*N* = 2)
		*Naming speed simple*					
		*RT*	1178 ± 23.85				
		*pt*	47136.69 ± 954.23				
		*errors*	1.15 ± 1.24				
		*Naming speed complex*					
		*RT*	1649.72 ± 39.05				
		*pt*	65988.27 ± 1562.16				
		*errors*	1.61 ± 0.30				
		*Symbol coding*					
		*RT*	1689.07 ± 52.57				
		*number of processed stimuli*	55.09 ± 1.83				
		*errors*	3.91 ± 0.47				
***Takahashi 2018***	Pre-treatment	*BPRS*	52.6 ± 13.2	Drug naïve	/	/	Antihistaminic, benzodiazepines, anticholinergics (*N* = na)
	After-treatment (2–8 weeks)		43.2 ± 14.6	Antipsychotic monotherapy	Na	3.2 ± 1.9 risperidone equivalents	
***Umesh 2018***		*PANSS*	40.25 ± 3.2	Antipsychotic mono- or polytherapy	FGA and/or SGA	443 ± 215.94 chlorpromazine equivalents	Na
		*SANS*	24.75 ± 4.70				
		*RSAS*	20.75 ± 1.41				
		*TEPS*	77.050 ± 1.877				
		*SPQ*	21.1 ± 1.74				
***Zeev-Wolf 2018***		*PANSS total*	44.1 ± 11.38	Antipsychotic mono- or polytherapy	FGA (*n* = 17) SGA (*N* = 13) FGA + SGA (*N* = 9)	Na	Na
***Lottman 2019***		*BPRS*	32.26 ± 9.82	Antipsychotic mono- or polytherapy	Drug naïve (*N* = 1) Aripiprazole (*N* = 2) Clozapine (*N* = 1) Risperidone (*N* = 16) Ziprasidone+clozapine (*N* = 1)	Na	No
		*SANS*	21.56 ± 17.81				
		*SAPS*	7.12 ± 11.77				
		*RBANS*	74.35 ± 15.31				
***Vignapiano 2019***		*PANSS*		Antipsychotic mono- or polytherapy	Na	603.64 ± 359.05 chlorpromazine equivalents	Na
		*Positive factor*	8.37 ± 4.52				
		*Disorganized factor*	8.60 ± 3.51				
		*BNSS*					
		*Avolition-apathy*	21.51 ± 9.02				
		*Expressive deficit*	11.64 ± 7.55				
		*MCCB*					
		*Speed of processing*	35.16 ± 9.40				
		*Attention/vigilance*	41.05 ± 10.64				
		*Working memory*	37.51 ± 11.14				
		*Verbal learning*	38.36 ± 10.95				
		*Visual learning*	33.36 ± 12.46				
		*Reasoning and problem solving*	39.83 ± 10.63				
		*Neurocognitive composite score*	33.89 ± 11.03				
***Alamian 2020***		*SANS*	3.96 ± 2.92	Antipsychotic mono- or polytherapy	Na	13.03 ± 13.80 Olanzapine equivalents	Na
		*SAPS*	3.72 ± 4.00				
***Freche 2020***		*SAPS*	11.0 ± 5.2	Antipsychotic mono- or polytherapy	FGA (*n* = 7), SGA (*n* = 12), both (*N* = 2)	Na	Na
		*SANS*	20.5 ± 3.6				
***Kim 2020***		*PANSS*	60.92 ± 21.71	Antipsychotic monotherapy (*N* = 33) Drug-free (*N* = 5)	Na	319.21 ± 307.81 chlorpromazine equivalents	Mood stabilizers (*N* = 6; valproate, lithium, lamotrigine, topiramate)
		*SOFAS*	64.54 ± 12.50				
		K-AVLT	8.37 ± 2.79				
***Krukow 2020***		*PANSS*	65.31 ± 11.18	Antipsychotic monotherapy	Aripiprazole (14.70%) Olanzapine (58.82%) Quetiapine (8.82%) Risperidone (17.64%)	4.73 ± 1.37 risperidone equivalents	No
***Lee 2020***		*PANSS*	62.3 ± 16.7	Na	/	/	/
		*TMT-A*	28.1 ± 13.4				
		*TMT-B*	72.6 ± 31.4				
		*CVLT, immediate recall*	9.8 ± 3.7				
		*CVLT, delayed recall*	10.1 ± 3.6				
***Soni 2020***		*SANS*	Na	Second generation antipsychotic	Na	Na	Na
		*SAPS*	Na				
***Tanaka-Koshiyama 2020***		*SAPS*	6.8 ± 4.0	Antipsychotics, non-specified	Na	Na	Anxiolytics (*N* = 25) Anticholinergics (*N* = 47)
		*SANS*	16.9 ± 3.8				
		*WRAT*	101.8 ± 10.6				
		*LN span*	12.8 ± 3				
		*LN sequencing*	10.0 ± 2.4				
		*WCST*	8.5 ± 7.2				
		*CVLT*	51.0 ± 10.0				
***Koshiyama 2021a***		*CVLT-II*	Na	Antipsychotic mono- (*N* = 115) orpolytherapy (*N* = 18) Drug free (*N* = 15)	FGA (*N* = 6) SGA (*N* = 109)	Na	Anxxiolytics (*N* = 26) Anticholinergics (*N* = 45)
		*Letter number sequencing*	Na				
		*SAPS*	6.7 ± 4				
		*SANS*	16.9 ± 3.8				
		*GAF*	41.1 ± 4.4				
***Koshiyama 2021b***		*GAF*	41.1 ± 4.3	Antipsychotic mono- or polytherapy (*N* = 127)	Na	Na	Anxiolytics (*N* = 25) Anticholinergics (*N* = 42)
		*SANS*	16.9 ± 3.8				
		*SAPS*	6.9 ± .41				
***Sun 2021***		*PANSS*		Drug-naïve or drug free (4 wks washout)	/	/	/
		*Positive*	15.80 ± 2.83				
		*Negative*	14.47 ± 7.65				
		*General*	32.07 ± 6.78				
***Yadav 2021***		*PANSS*	61.66 ± 15.01	Drug free	/	/	No
***Gordillo 2022***		*SANS*	10.1 ± 5.2	Antipsychotic mono- or polytherapy (*N* = 106) Drug free (*N* = 15)	Na	561.1 ± 389.4 chlorpromazine equivalents	Na
		*SAPS*	8.6 ± 3.2				
***Tagawa 2022***		*PANSS*	63.8 ± 22.6	Antipsychotic mono-or polytherapy (*N* = 24) Drug free (*N* = 5)	Na	451 ± 414.7 chlorpromazine equivalents	Antidepressant (*N* = 2) Benzodiazepines (*N* = 13)
***Ibanez-Molina 2023***		*Na*	/	Antipsychotic monotherapy	SGA	818.18 ± 407.75 chlorpromazine equivalents	Antidepressant, unspecified (*N* = 1)
***Jacob 2023***		*PANSS*	65.43 ± 34.06	SGA (*N* = 39), FGA (*N* = 4), FGA + SGA (*N* = 1), untreated (*N* = 13)	Na	Na	Na
***Yeh 2023***		*PANSS*	71.57 ± 9.08	Antipsychotic mono-or polytherapy	Na	603.64 ± 359.05 chlorpromazine equivalents	Na
		*PSP global score*	53.13 ± 10.42				
		*CPT-II*					
		*d’*	0.74 ± 0.55				
		*OM*	14.33 ± 26.41				
		*COM*	14.94 ± 9.29				
		*PER*	3.79 ± 6.94				
		*HRT*	467.25 ± 102.64				
		*HRTSE*	9.73 ± 8.11				
		*VAR*	17.23 ± 17.72				
		*HRTBC*	0.01 ± 0.03				
		*HRTISIC*	0.06 ± 0.04				
		*CTT1*	57.11 ± 23.39				
		*CTT2*	104.99 ± 33.96				
		*WCST non-perseverative error*	18.36 ± 18.62				
		*TOL accuracy*	4.04 ± 2.27				
		*TOL time*	235.31 ± 92.13				
***Chang 2024***		*PANSS*		Antipsychotic polytherapy (at least two)	Na	>600 chlorpromazine equivalents	Na
		*Total*	91.0 ± 12.41				
		*Positive*	25.73 ± 3.03				
		*Negative*	26.07 ± 5.48				
		*General*	39.20 ± 7.48				
		*Psychotic symptoms rating scale*					
		*Total*	53.00 ± 9.52				
		*Auditory hallucinations*	33.07 ± 6.46				
		*Delusions*	19.93 ± 3.84				

*BACS* brief assessment of cognition in schizophrenia, *BNSS* brief negative symptoms scale, *CAARMS* Comprehensive Assessment of At Risk Mental States, *COM* commission error, *CPT-II* Connors’ continuous performance test, 2nd edition, *CTT* color trail test, d’ detection, *CVLT-II* California verbal learning test II, *FGA* first generation antipsychotic, *GAF* global assessment of functioning, *HRT* hit reaction time, *HRTSE* HRT standard error, *VAR* variability, *HRTBC* HRT block change, *HRTISIC* HRT interstimulus interval change, *K-AVLT* Korean auditory verbal learning test, *MCCB* MATRICS cognitive consensus batter, *OM* omission error, *PANSS* positive and negative syndrome scale, *PER* perseveration, *PSP* personal and social performance scale, *RBANS* Repeatable Battery for Assessment of Neuropsychological Status, *RSAS* revised social anhedonia scale, *SANS* scale for the assessment of negative symptoms, *SAPS* scale for the assessment of positive symptoms, *SPQ* schizotypal personality questionnaire, *TEPS* temporal experience of pleasure scale, *TMT* trail-making test, *TOL* Tower of London, *WCST* Wisconsin card sorting test, *WRAT* Wide Range Achievement Test.

**Table 3:** EEG and MEG methods.

Reference	Recording	Sampling rate	Filtering	Artifact rejection	Epochs	Data normalization	Source location	Analysis
***Rutter 2009***	MEG, 275 ch, Eyes closed, 4’	600 hz	0.61–150 hz, 60 hz notch	First and last 10” rejected	Na	Constant noise estimate; absolute/relative power use: Na	Synthetic aperture magnetometry (SAM); constant noise estimation normalization	SAM Power spectra (source level)
***Venables 2009***	EEG, 27 ch, 3’ eyes open and 3’ eyes closed	500 hz	0.05–100 hz, 60 hz notch	Epochs exceeding ± 200 mV, 2 hz high pass, PCA	4” *N* = Na	Baseline correction; use of absolute power	Na	Rectified frequency amplitude (sensor level)
***Kikuchi 2011***	EEG, 17 ch, 3’ with eyes closed	200 hz	1.5–60 hz	Manual visual inspection	2.56” *N* > 15	averaging over the all epochs; log transformation	Na	Omega complexity and Local Complexity Differentials (sensor level)
***Hanslmayr 2012***	EEG, 61 ch, 4’, eyes open	500 Hz	0.1–250 hz, 50 hz notch	Manual visual inspection, ICA	2” *N* = 115	Use of relative power	Dynamic imaging of coherence sources (DICS)	Mean power levels (source level)
***Andreou 2014a***	EEG, 64 ch, 5–10’ with eyes closed	1000 hz	0.1–70 hz	ICA	2” *N* = 198 ± 40.4	Na; use of relative power	eLORETA	Power envelopes correlates (source and sensor level)
***Andreou 2014b***	EEG, 64ch, 5–10’ with eyes closed	1000 hz	0.1–70 hz	ICA	2” *N* = 212 ± 52.3	Na; use of relative power	eLORETA	Multivariate interaction measure (source and sensor level)
***Garakh 2014***	EEG, 19 ch, 100” with eyes closed	Na	70 hz	custom designed multiple-source eye correction method (Novototskii-Vlasov et al., 2007), visual inspection	10–15”	.spectral power logarithmic Transformation, use of relative power	Na	Mean spectral power (μV^2^/hz), (sensor level)
***Kam 2014***	EEG, 32 ch, 3’, eyes closed	1000 hz	High pass 0.5 Hz and 60 hz notch	Exclusion of activity >100 V; algorithm (Gratton 1983) for eye movement and blink removal; ICA	2.048”; at least 50” of artefact free data	Mean absolute power for each frequency band,logarithmic transformation	Na	Mean absolute power spectra, coherence using BrainVision Analyzer (sensor level)
***Kim 2014***	MEG, 306 ch, 150 s, eyes open	1001 hz	0.1–200 hz; notch na	Manual removal based on visual inspection	2.56” *N* = na	No; use of absolute power	sLORETA	Absolute current estimates, frequency spectrum, coherence estimates (source level)
***Di Lorenzo 2015***	EEG, 37 ch, 3’ with eyes closed	1024 hz	1–100 hz, 60 hz notch	Semiautomatic removal of artifacts, ICA	2” *N* = na	Na; use of absolute power	eLORETA using Montreal neurological institute space (MNI)	Spectral time series of centroid voxel for each ROI, eLORETA connectivity algorithm (source level)
***Hirano 2015***	EEG, 71 ch, not specified duration and eyes open/closed conditions	512 hz	0.1–100 hz	Exclusion of activity>200 microV and variation >90 microvolt, ICA	1” *N* = 139 + 27	Na, use of absolute power	Single epoch source dipole; BESA	Time-frequency and power spectra analysis using generalized Morse wavelet transform; debiased phase amplitude coupling (source level)
***Mitra 2015***	EEG, 192 ch, 3’, eyes closed	512 HZ	0.1–120 hz, 50 hz notch	Visual inspection	30”	Recomputing with common average reference; use of absolute power	Na	Averaged power spectra (sensor level)
***Tikka 2015***	EEG, 192 ch, 10’ eyes closed	512 hz	30–100 hz, 50 hz notch	Visual inspection	Na; at least 60” of clean EEG	Recomputing with common average reference; Log transformation; use of absolute power	Na	Spectral power with Welch periodogram, cross spectral coherence (sensor level)
***Ramyaed 2016***	EEG, 19 ch, 20’ eyes closed	250 hz	1 hz, 50 hz notch	Visual inspection + ICA	2” *N* = 638	Na; use of absolute power	eLORETA, statistical nonparametric mapping	Current source density analysis, lagged phase synchronization (source level)
***Umesh 2016***	EEG, 192 ch, 10’ with eyes closed	512 hz	0–120 hz	Visual inspection	Na; 60” total recording	Log transformation, application of Fisher Z, use of absolute power	Na	Spectral power and cross-spectral coherence with the Welch averaged periodogram method (sensor level)
***Won 2017***	EEG, 21 ch, 4’, eyes closed	1000 hz	1 hz high pass, 60 hz notch	Visual inspection + ICA	1”, at least 2’ of clean EEG	detrending to remove the DC component; removal of outliers for *P* < 0.05; use of absolute power	Na	Absolute spectral powers; Cohen synchronization index (sensor level)
***Arikan 2018***	EEG, 19 ch, 3’, with eyes closed	500 hz	0.15–70 hz	Visual inspection	Na	Na; use of absolute power	Na	Averaged spectral power (sensor level)
***Baradits 2018***	EEG, 256 ch, 2’ with eyes closed	512 hz	0.1–100 hz, 48–52 hz notch	Manual visual inspection + ICA using the ADJUST toolbox	2” *N* = na	Log10 transformation; use of absolute power	Na	Absolute power based on the Welch’s method; spectral centroid calculation (sensor level)
***Grent-t’-Jong 2018***	MEG, 248 ch, 5’ with eyes open	1017.25 hz	0.5–150 hz, 50 hz notch	Visual inspection + ICA	1” *N* = 240	Data rescaled per trial and channel, use of absolute power	Dynamic imaging of coherence sources (DICS) beamforming	Power spectra (source level)
***Hirano 2018***	EEG, 71 ch, not specified duration and eyes open/closed conditions	512 hz	Na	ICA with the debiased phase amplitude coupling procedure	0.5” *N* = na	Normalization of the dPAC with z scores; use of absolute power	Single epoch waveform for each source dipole using the brain electric source analysis (BESA)	Debiased phase-amplitude coupling measure by von-Driel Morse wavelet (source level)
***Jonak 2018***	EEG, 21 ch, 10’ recording with eyes closed	512 hz	0.5–70 hz, 50 hz notch	Visual inspection	8” *N* = 25	Na	Na	Phase lag index, graph analysis with a minimum spanning tree (sensor level)
***Krukow 2018***	EEG, 21 ch, 10’ with eyes closed	500 hz	0.5–50 hz,	Visual inspection	8.19” *N* = 8	Na	Na	Phase lag index (sensor level)
***Takahashi 2018***	EEG, 16 ch, 10–15’ with eyes closed	200 hz	1.5–60 hz	Visual inspection; elimination of initial and final epochs	5” *N* = 12	Use of relative power	Na	Phase lag index, spectral power, coherence (coherence matrices, Hilbert transform), node degree calculation (sensor level)
***Zeev-Wolf 2018***	MEG, 248 ch, 2’ with open eyes	1017 hz	0.1–100 hz	Visual inspection + ICA	20”, 120 epochs	Na; use of absolute power	Cross-spectral density matrix, construction of a shell brain model	Averaged power spectra (source and sensor level)
***Lottman 2019***	MEG, 148 ch, 5 min with eyes closed	1000 hz	0.1–200 hz, Notch filter 60, 120 hz	Visual inspection + ICA	Na	standardizing the Hilbert Data covariance matrices were regularized using a median eigenvalue approach; envelope with a 1/frequency compensation; Absolute power values of envelopes	Linearly constrained minimum variance beamformer	Hilbert envelopes’ values computation; pairwise Pearson’s correlations between RSN time courses (source level)
***Vignapiano 2019***	EEG, 29 ch, 5’ with eyes closed	512 hz	0.15–70 Hz	Visual inspection + ICA	2”, at least 50% of epochs recorded to be included	Na; use of absolute power	Na	Square root of averaged spectral power (sensor level)
***Alamian 2020***	MEG, 275 ch, 5’ with eyes open and 5’ with eyes closed	1200 hz	0.1–150 hz; 60, 120, 180, 240, 300 hz notch	Visual inspection + ICA	Na	Na	NeuroPycon pipeline with minimum norm estimates	Detrended fluctuation analysis; support vectoring machine on long range temporal correlations (source level)
***Freche 2020***	EEG, 64 ch, 260 ± 60” with eyes open	1024 hz	1–150 hz, 50 hz notch	Visual inspection + ICA; elimination of initial and final epochs	Na 180” total	data normalized using the quartile-based coefficient of variation; use of relative power	Na	Relative power and power spectra density (sensor level)
***Kim 2020***	EEG, 62 ch, 5’ with eyes closed	1000 hz	1–100 hz	Visual inspection	2” *N* = 30	Use of relative power	Depth-weighted minimum L2 norm estimator	Current source densities; phase locking values; graph theory based network analysis (source level)
***Krukow 2020***	EEG, 21 ch, 15’ with eyes closed	250 hz	0.5–70 Hz, 50 Hz notch	Manual visual inspection	16” *N* = 45	Na, use of absolute power	eLORETA using Montreal neurological institute space (MNI) and statistical nonparametric mapping	Lagged phase synchronization (source level)
***Lee 2020***	EEG, 128 ch, 2–5’, with eyes closed	1000 hz	0.5–100 Hz	Manual visual inspection + ICA	4” *N* = 25	Na; use of absolute power	sLORETA	Basic finite impulse filter, modulation index for theta-gamma coupling (source level)
***Soni 2020***	EEG, 128 ch, 5–6’ with eyes closed	1000 hz	1–100 hz	Manual visual inspection	1” *N* = 20	Na; use of absolute power	Equivalent current dipole, MNI brain model	Power spectra density, linear coherence analysis (source level)
***Tanaka-Koshiyama 2020***	EEG, 40 ch, 328 s, eyes open	1000 hz	0.5–100 Hz	Visual inspection + EEGLAB plugin “clean raw data” + ICA	Na	Na	Equivalent current dipole with fieldtrip function	Grand averaged spectral power (μV^2^/hz) (sensor level)
***Koshiyama 2021a***	EEG, 40 ch, 3 min with eyes open	1000 hz	0.5–100 Hz	EEGLAB plugin “clean rew data” including artefact subspace reconstruction	5” *N* = Na	Na	Equivalent current dipole, fieldtrip function	Power spectra density using Welch method; phase discontinuity index using wavelet transform data (source level)
***Koshiyama 2021b***	EEG, 40 ch, 3 min with eyes open	1000 hz	0.5–100 Hz	EEGLAB plugin “clean rew data” including artefact subspace reconstruction	Na	Na	Equivalent current dipole using fieldtrip function	Phase amplitude coupling toolbox for EEG lab, using Hilbert transform (source level)
***Sun 2021***	EEG, 64 ch, 7 min with eyes closed	500 hz	0.1–100 hz	Na	Na	Na	Na	Binarized, weighted network analysis evaluating clustering coefficient and path length (sensor level)
***Yadav 2021***	EEG, 192 ch, 10 min with eyes closed	Na	0.1–120 Hz	Visual inspection	60” *N* = Na	Log transformation; use of absolute power	Na	Spectral power with Welch’s averaged periodogram (sensor level)
***Gordillo 2022***	EEG, 64 ch, 5 min with eyes closed	2048 hz	0.1–100 hz; 50 hz notch	Visual inspection + ICA	2” *N* = 30–40	Log transformation; use of absolute power	LORETA	Time domain amplitude features, range EEG, Hjorth parameters, spectral amplitude, modulation index, fractal dimension, hurst exponent, detrended fluctuation analysis, life and waiting times, entropy in the time domain, complexity measures, recurrent quantification analysis, microstate parameters, directed transfer function, instantaneous and lagged phase synchronization, network analysis, partial least square correlation (sensor and source level)
***Tagawa 2022***	MEG, 306 ch, 7 min with eyes open	Na	0.1–400 hz, 50 hz notch	Oversampled temporal projection; signal space separation; ICA	Na	0–1 rescaling; use of absolute power	Minimum norm estimates	Graph analysis on orthogonalized amplitude envelope correlations parameters: degree centrality, clustering coefficient, global efficiency, local efficiency, small worldness (source level)
***Ibanez-Molina 2023***	EEG, 31 ch, 5 min with eyes closed	1000 hz	Na	Visual inspection + ICA	Na	Na	Na	Mutual information of multiple rhythm (MIMR): sample entropy and phase amplitude coupling (Hilbert transform) (sensor level)
***Jacob 2023***	EEG, 32 ch, 6 min eyes open	Na	0.5–100 hz	artifact subtraction, canonical correlation analysis, semi-automatic hearthbeat detection algorithm, ICA	2” *N* = Na	Na; use of absolute power	Na	Power spectra, aperiodic power (sensor level)
***Yeh 2023***	EEG, 32 ch, 5 min eyes open and 5 min eyes closed	4000 hz	0.5–100 hz	Visual inspection + ICA	4” *N* = 25	Na, use of absolute power	eLORETA	Lagged phase synchronization (source level)
***Chang 2024***	EEG, 64 ch, 4 min eyes open and 4 min eyes closed	1000 hz	1–200 hz, 50 hz notch	ICA; exclusion of variations > ±100 µV	2” *N* = Na	Na	Na	Weighted Phase lag index with network analysis (node degree, global efficiency, local efficiency, betweenness centrality, clustering coefficient) (sensor level)

*ANCOVA* analysis of covariance, *ANOVA* analysis of variance, *LORETA* low-resolution tomography analysis, *MANOVA* multivariate analysis of variance, *MNI* Minnesota neuroscience institute, *TANOVA* topographic analysis of variance, *TANCOVA* topographic analysis of co-variance.

Thirty-six studies used EEG, 7 used MEG; the total sample size was 2133 patients, ranging from 11 to 185 subjects per study and 37.6% of the participants were females. The mean age was 33.8 ± 9.2 years. Forty-one studies included a control group, for a total of 2040 participants (38% of females) with a mean age of 32.5 ± 7.8 years.

The AMSTAR checklist^36^ was used to foster the quality of this review and the meta-analysis. The included studies were described in detail (in the text or in the tables) concerning population, intervention, comparators (i.e., control group, different diagnostic groups, same patient on different time points), outcomes and research design. Methods were established prior to the review, and all studies suitable to investigate the variable of interest (i.e., gamma-band oscillation related to schizophrenia and its clinical features) were included. Study selection and data extraction were performed in duplicate. The exclusion of initially retrieved studies was explained. Funding and authoring were clearly reported.

The Newcastle Ottawa scale for case-control studies was used to rate the quality of each included study, as detailed in Supplementary Table S1. The quality was overall good, with an average score of 5.78 ± 1.4, in a reduced scale including only 6 out of 8 items.

### Overview

Table 4 comprehensively reports the findings of each study in detail, and Supplementary Table S2 reports region-specific positive findings specific for each brain area. In the following sections, a synthesis of results is offered, delineating common patterns emerging across studies. Different aspects of gamma dynamics were collected in sections on power spectra, functional connectivity and phase-amplitude coupling. In each section, sub-headings are used to separate findings on the difference between patients and controls, and on the relationship of gamma frequencies with clinical features. In each sub-heading, we first report the number of studies observing positive, negative or null associations, then, the brain area, the lateralization, the gamma subband and the source localization method. In the relative section, functional connectivity measures are reported. Then, differences recording, preprocessing and analysis methods are reported in the text, and extensively described in Tables 5 and 6 (for studies on power spectra and functional connectivity, respectively). Secondary findings related to the topic of each section are reported at its end.

**Table 4:** Summary of findings in patients within the schizophrenia spectrum.

***Rutter 2009***	• Reduced gamma1 and gamma2 and gamma3 power in bilateral precuneus, cuneus, posterior cingulate cortex, middle occipital gyrus and cingulate gyrus, compared to healthy controls • Compared to healthy controls, unaffected siblings of patients with schizophrenia showed a reduced gamma1, gamma2, gamma 3 power in the following left hemisphere areas: middle occipital gyrus, lingual gyrus, cuneus, inferior occipital gyrus, fusiform gyrus, inferior temporal gyrus and middle temporal gyrus.
***Venables 2009***	• No gamma1 power spectra differences between patients with schizophrenia, fist degree relatives and controls • First-degree relatives exhibited an increased in gamma1 amplitude at bilateral temporal sites compared with controls, in the eyes-open condition.
***Kikuchi 2011***	• Higher values of gamma1 omega complexity, compared to healthy controls; • The contribution of the right frontal area to gamma1 omega complexity was higher in schizophrenia compared to controls; • No correlation between severity of symptoms (BPRS total score) and gamma1 spectral power;
***Hanslmayr 2012***	• No differences between patients and controls in resting state gamma1 power
***Andreou 2014a***	• Increased mean gamma1 connectivity compared to controls in the left rolandic operculum (single region level); • Increased mean gamma1 connectivity between the left parietal and temporal regions with homolateral lateral and orbital frontal areas; minor contributions from the same regions in the contralateral hemisphere; • Patients with high-symptoms (in the disorganization and positive symptoms factors) showed a significantly lower gamma1 connectivity in the network described above, compared to patients with low-symptoms and to healthy controls
***Andreou 2014b***	• No differences between patients and controls in resting state gamma1 band connectivity
***Garakh 2014***	• No differences between patients and controls in resting state gamma1 power; • No correlation between overall severity of symptoms and gamma1 power
***Kam 2014***	• Increased whole-brain gamma1 power in bipolar disorder compared to schizophrenia; • No significant differences in gamma1 power spectra when comparing patients with schizophrenia and controls; • No significant differences in gamma1 power short- and long-range connectivity when comparing patients with schizophrenia and controls
***Kim 2014***	• Positive correlation between the PANSS positive symptoms’ subscale and the gamma1 power of the bilateral medial prefrontal cortex, no association with the posterior cingulate cortex • Reduced gamma1 coherence between the medial prefrontal cortex and the posterior cingulate cortex in patients vs controls; • No significant differences in gamma1 power spectra when comparing patients with schizophrenia and controls
***Di Lorenzo 2015***	• Using phase lagged phase synchronization, gamma1 and gamma2 connectivity was increased between right occipital-right prefrontal, right occipital-right parieto-temporal, and right occipital-right cingulate pairs, compared to healthy subjects; • Patients with a duration of disease inferior to 5 years showed a whole-brain increase in gamma1-gamma2 connectivity compared to healthy subjects and to patients with a duration of disease superior to 5 years; • In a region by region analysis, patients with a duration of disease superior to 5 years displayed a decreased connectivity between prefrontal and temporo-parietal regions, an increased connectivity between occipital and right cingulate cortex and between occipital and right temporal cortex, compared to healthy subjects, in the gamma1-gamma2 range.
***Hirano 2015***	• In the auditory cortex, no significant differences in whole gamma power (i.e 30–100 hz) when comparing patients with schizophrenia and controls
***Mitra 2015***	• Gamma1 power was increased vs controls in the right frontal and left occipital cortex; • Gamma2 power was increased vs controls in the left frontal area; • Gamma3 power was increased vs controls in the bilateral parietal, left temporal and central regions; after correction for multiple testing, differences persisted only for the left parietal and temporal gamma3 power, where power was increased
***Tikka 2015***	• In gamma1, power was increased in bilateral frontal, right parietal, bilateral temporal, bilateral occipital and central cortex, compared to healthy controls; • In gamma1, power in bilateral frontal, left temporal, right temporal left occipital and central regions was increased in patients with schizophrenia compared to first-degree relatives; • In gamma1, first degree relatives had an increased right temporal power than healthy controls; • In gamma2, power was increased in bilateral frontal, bilateral central, bilateral parietal, bilateral temporal and right occipital regions compared to healthy controls • In gamma2, power in the left occipital region was higher in patients than in first-degree relatives • In gamma2, first degree relatives had higher gamma power compared to healthy subjects in the right parietal and right temporal regions • In gamma3, power was increased in the right frontal, right occipital, central, right parietal, right temporal and all the left regions in patients with schizophrenia compared to healthy subjects • In gamma3, power was increased in right frontal, right occipital and central regions in patients compared to first degree relatives; • In gamma3, first degree relatives had an increased power the right parietal and temporal regions compared to healthy controls; • Bilateral frontal bilateral parietal, bilateral temporal, bilateral occipital and central regions, compared to both healthy controls and first-degree relatives; • Gamma1 power in the left temporal region had a negative correlation with the PANSS general psychopathology sub-scale; PANSS total score correlated negatively with the left temporal gamma1 and gamma2 power, compared to healthy subjects and first-degree relatives; • Increased gamma1 intrahemispheric spectral coherence: in gamma1 in the left fronto-temporal cortex, compared to healthy subjects and first-degree relatives; • Increased gamma2 intrahemispheric spectral coherence in the right parietal and occipital regions, compared to first degree relatives; • Increased gamma3 intrahemispheric spectral coherence in the right parietal and occipital regions, compared to healthy subjects and first degree relatives • No differences in interhemispheric gamma spectral coherence between patients, first degree relative and healthy controls
***Ramyaed 2016***	• The highest amount of oscillatory activity in the first episode of psychosis was in gamma-band (only gamma1 was evaluated), differently from healthy controls, for whom it was in alpha2 band; • Increased gamma1 activity in the left medial frontal gyrus compared to healthy controls; • No correlation between severity of symptoms and gamma spectral power
***Umesh 2016***	• Lower gamma3 power in midline, left parietal, right temporal and occipital regions compared to control subjects, in both patients with schizophrenia and unaffected first degree relatives; • Negative correlation between right temporal gamma3 power and social anhedonia; • Lower gamma3 coherence over the right temporal and right fronto-occipital regions (not surviving correction for multiple comparisons);
***Won 2017***	• Higher values of theta-gamma (including gamma1 and gamma2 sub-bands) coupling in patients compared to control subjects, at the whole brain level; for the discrimination of the two accuracy was 92.5%, sensitivity 88%, specificity 83%.
***Arikan 2018***	• Increased gamma1 power at the electrode location C3, with a trend also in Cz (analysis was restricted to the electrodes for whom a significant relationship with insight was detected), compared to healthy subjects; • Gamma1 power was significantly correlated to insight (i.e., the capacity of the patient to evaluate its own psychological status) at F3, C3, and Cz electrodes location; • No significant relationships of gamma1 power with negative symptoms severity
***Baradits 2018***	• Increased gamma1 power in a fronto-central cluster (29 electrodes) and in a posterior cluster (49 electrodes); • A higher symptoms severity was related to an increased gamma1 power in the left occipital cortex. This difference was valid for 13 channels for PANSS total score, for 11 channels for the negative symptoms sub-scale, and for 15 channels for a hostility factor; • No differences in the distribution of gamma1 power between patients and controls.
***Grent-t’-Jong 2018***	• First episode of schizophrenia patients had a decreased prefrontal gamma1 power and an increased occipital gamma1 power, compared to healthy controls; • Chronic schizophrenia patients showed a decreased gamma1 in frontal, temporal and sensorimotor areas, compared to healthy controls; • Gamma2 and gamma3 power were increased in posterior regions for the first-episode group and decreased in frontal regions in both first-episode and chronic patients; • The brief assessment of cognition in schizophrenia scale (BACS) composite score and the Comprehensive Assessment of At-Risk Mental States (CAARMS) negatively correlated with whole band gamma power (with a stronger relationship for gamma3) in posterior areas in first episode of schizophrenia patients, while frontal and central areas showed an opposite relationship; • In chronic schizophrenia, BACS and PANSS total score positively correlated with gamma-power, especially in gamma1;
***Hirano 2018***	• No significant differences in whole gamma power spectra (30–100 hz) when comparing patients with schizophrenia and controls; • Phase amplitude coupling of gamma oscillations with delta, theta and alfa frequencies was not different in patients with schizophrenia compared to healthy subjects; • Theta gamma phase amplitude coupling was lateralized to the left in control subjects and not lateralized in patients.
***Jonak 2018***	• No differences in the gamma1 phase lag index of first episode and chronic patients; • Increased gamma1 hierarchy in chronic schizophrenia compared to first-episode of psychosis; • Gamma1 hierarchy was positively related to number of hospitalizations and duration of illness; • A topological analysis demonstrated that first episodes of schizophrenia had a configuration with more leaves, compared to chronic patients. For first-episode, the nodes with the highest betweenness centrality values were placed in the right and left temporal left lateral prefrontal cortex (F7, F8) and in the right posterior temporal area (T6). Smaller hubs in the right temporal lobe (T4), the parietal lobe (P3, P4), the right occipital pole (O2), and on the mid-point of the frontal lobe (tFz); • The main hubs for the chronic group in the gamma band were in the midline (Pz), and in the left posterior temporal area (T5). Minor hubs were in temporal lobe (T3, T4), left sensorimotor cortex (C3), right posterior temporal area (T6), right occipital pole (O2), left frontal lobe (Fp1) and right middle frontal area (F4); • No significant differences in gamma1 power when comparing patients and controls
***Krukow 2018***	• Gamma1 phase-lag index was significantly lower in patients compared to controls for right fronto-occipital, left temporo-central areas and overall, but higher for right temporo-parietal and temporo-occipital areas; • No association of gamma1 connectivity with cognitive processing speed; • No association of gamma1 connectivity with arithmetic tasks
***Takahashi 2018***	• Reduced phase lag index in gamma1-gamma2 band across widespread scalp regions; • No significant group differences in gamma1 and gamma2 band between patients with schizophrenia and healthy controls
***Zeev-Wolf 2018***	• No significant differences in gamma1 and gamma2 power spectra when comparing patients with schizophrenia and controls • No correlation between severity of symptoms and gamma1-gamma2 power
***Lottman 2019***	• No significant differences in gamma1 short- and long-range connectivity between patients with schizophrenia and controls
***Vignapiano 2019***	• No significant differences in gamma1 and gamma2 power spectra when comparing patients with schizophrenia and controls; • No correlation between severity of symptoms and gamma1-gamma2 spectral power, in the positive, avolition-apathy and diminished expression domains;
***Alamian 2020***	• With eyes closed, higher gamma1-gamma2 spectral amplitude in orbital sulci and gyri, parahippocampal gyri, temporal poles, left superior temporal sulcus and left temporal gyrus. Gamma-band amplitude of left caudate amygdala and hippocampus significantly discriminated patients and controls. The highest discrimination occurred in the left temporal pole (accuracy 84%). • In eyes open conditions, increased gamma1-gamma2 oscillatory amplitude, in left rectus gyrus, right orbital gyrus, orbital sulci and left parahippocampal gyrus. Significant classification of patients and controls in amygdala, hippocampus and cerebellum; • No correlation between severity of symptoms and gamma1-gamma2 spectral power; • No differences in long-range temporal correlation in the gamma range, between patients and controls
***Freche 2020***	• Increased gamma1 power compared to healthy controls in bilateral central and prefrontal-frontal electrodes
***Kim 2020***	• No significant differences in gamma1 power spectra when comparing patients with schizophrenia and controls
***Krukow 2020***	• decreased gamma1 power in the postcentral gyrus of the right hemisphere in patients vs controls • Gamma1 connectivity within the temporal and parietal lobes of the right hemisphere (connectivity of right middle frontal gyrus with the inferior parietal lobule and with the post central gyrus) was significantly weaker, compared to healthy controls. • No association of gamma1 functional connectivity with performance initiation (i.e., a cognitive activity related to transition from resting-state to engagement in task execution)
***Lee 2020***	• The modulation index of theta-gamma1 coupling in patients with a first episode of schizophrenia was higher than that in healthy controls in the posterior cingulate cortex (after correction for multiple comparisons); • Theta-gamma1 coupling values in posterior cingulate cortex correlated with trail-making test A and B, immediate recall and delayed recall tests;
***Soni 2020***	• Patients and their first-degree relatives had a higher whole-band gamma power (30–100 hz) in the left parahippocampal gyrus, compared to healthy controls; however, in a cluster located in the left deep parahippocampal gyrus, patients had less gamma-power compared to first-degree relatives and controls; • Compared to healthy controls, whole-band gamma functional connectivity was reduced between left parahippocampal gyrus and left insula; cingulate gyrus and left posterior cingulate cortex; left insula and right insula; cingulate gyrus and left anterior cingulate cortex; • Compared to healthy first-degree relatives, whole-band gamma functional connectivity was reduced between left parahippocampal gyrus and left insula, cingulate gyrus, left anterior cingulate; between left deep parahippocampal gyrus and left anterior cingulate; left insula and right insula, cingulate gyrus and left posterior cingulate; left anterior cingulate cortex and cingulate gyrus.
***Tanaka-Koshiyama 2020***	• Whole brain gamma1 power was increased in patients compared to controls; • An analysis restricted to patients not prescribed with anxiolytics and anticholinergics showed a relationship between gamma1 power and verbal learning
***Koshiyama 2021a***	• Higher gamma1 power in right supplementary motor area, right middle temporal gyrus, left middle temporal pole, left fusiform gyrus and near the right calcarine sulcus, compared to controls; • No differences in the phase discontinuity index (i.e., a metric used in the analysis of electroencephalographic (EEG) signals to quantify irregularities or discontinuities in brain wave patterns) between patients and controls • No association of gamma power spectral density and the california verbal learning test II score and the letter number sequencing test (for working memory)
***Koshiyama 2021b***	• Decreased delta-band phase synchronization with gamma1 and gamma2 power in the frontocentral, right middle temporal, left temporoparietal regions; • Increased delta-band phase synchronization with gamma1 and gamma in the left parietal regions, compared to healthy subjects; • The mean resultant vector length (i.e., a strength of delta and theta phase synchronization related to gamma1 and gamma2 power) in patients was significantly shorter in Fz, T8 and Tp9, and longer in P7, compared to healthy controls
***Sun 2021***	In patients with auditory verbal hallucinations gamma1 graph’s clustering coefficient was higher compared to controls.
***Yadav 2021***	• Increased gamma1 power compared to controls in: in bilateral frontal; bilateral parietal; left temporal; bilateral occipital; midline cortex; • Increased gamma2 compared to controls in: right parietal; left temporal; bilateral occipital and midline cortex • No significant differences between patients and controls in the gamma3 band • Duration of illness negative correlated with bilateral frontal gamma1 and gamma3 spectral power, and to gamma3 in the right temporal region • A linear regression analysis showed that the duration of illness was a predictor of gamma1 power in the bilateral frontal region, and for gamma3 in the bilateral frontal and right temporal regions in the first episode of psychosis
***Gordillo 2022***	• Compared to healthy controls, the following gamma1 and gamma2 related features were increased in patients with schizophrenia: clustering coefficient of the lagged phase synchronization at source level; Higuchi’s Fractal Dimension; kurtosis amplitude; modulation index alpha-gamma; modulation index delta-gamma, node strength and the imaginary part of coherency at electrode level; node strength of the instantaneous phase synchronization at source level, node strength of the lagged phase synchronization at source level in the gamma band; • Compared to healty controls, the following gamma1 and gamma2 related features were reduced in patients with schizophrenia: betweenness centrality of the lagged coherence at source level; the coefficient of variation of the gamma amplitude, the beta-gamma modulation index, the spectral entropy. • None of these features was related to severity of disease
***Tagawa 2022***	• Lower clustering coefficient (with a 20% threshold) and local efficiency in gamma1 and gamma2 range, compared to healthy controls
***Ibanez-Molina 2023***	• Lower alpha-gamma1 coupling in the whole brain, compared to healthy controls; • Increased theta-gamma1 coupling in bilateral prefrontal areas compared to controls
***Jacob 2023***	• No significant differences in gamma1 power spectra when comparing patients with schizophrenia and controls
***Yeh 2023***	• Attention had a significant positive association with gamma1 lagged phase synchronization between the right hemispheric cuneus and the transverse temporal gyrus and the right hemispheric cuneus and the superior temporal gyrus. The former association accounted for 29% of attention score variance, the latter for 25% of it.
***Chang 2024***	A Weighted phase lag index model was used to construct the brain network. • At the whole brain level, clustering coefficient, global efficiency, node degree, local efficiency and betweenness centrality, in eyes-open and eyes-closed conditions were increased compared to healthy controls, in the gamma1-gamma2 range; • In the temporal pole mean degree and mean betweenness centrality in eyes-open and eyes-closed conditions were higher compared to healthy controls, in the gamma1-gamma2 range; • in frontal pole mean degree and mean betweenness centrality in eyes-open and eyes-closed conditions were lower compared to healthy controls, in the gamma1-gamma2 range; • A positive correlation was found between symptoms severity and local-efficiency in eyes closed conditions, in the gamma1-gamma2 range; • A positive correlation was found between symptoms severity and node degree of frontal lobes in eyes-open conditions, in the gamma1-gamma2 range.

**Table 5:** Clinical, preprocessing and analysis factors potentially implicated in heterogeneity of findings for power spectra (patients vs controls).

Parameter	Studies with increased gamma power (*N* = 13)	Studies with decreased gamma power (*N* = 3)	Studies with mixed results (*N* = 2)	Studies with no group differences (*N* = 14)
**Total sample size**	716	92	55	652
**Age**	35.8 [30 | 44.8]	30 [25.5 | 30.6]	32 [29.5 | 34.5]	37 [27.7 | 42.4]
**Sex (F)**	29% [18.75 | 34.55]	29% [14.5 | 42]	27.5% [26.25 | 28.75]	30.5% [19.25 | 56]
**Duration of illness (months)**	124.8 [61 | 244.75]	12.7	Na	145 [101 | 196]
**Severity of disease (PANSS total score)**	84 [83 | 87.6]	40 (Na: 2)	61.6 [59 | 64.25]	63.75 [56.4 | 71.4]
**Medication status**	Medicated: 7	Medicated: 2	Medicated:1	Medicated: 7
	Unmedicated: 4	Na: 1	Unmedicated: 1	Mixed^a^: 7
	Mixed^a^: 2			
**Recording method**	EEG: 11	EEG: 2	EEG: 0	EEG: 12
	MEG: 2	MEG: 1	MEG: 2	MEG: 2
**Number of channels/sensors**	128 [40 | 192]	192 [106 | 233]	248	46 [27 | 69]
**Recording duration**	5 [3 | 5.5]	10 [7 | 12]	5	4.5 [3 | 6.5]
**Eyes open/eyes closed**	Open: 4	Open: 0	Open: 2	Open: 4
	Closed: 9	Closed: 3	Closed: 0	Closed: 8
	Na: 0	Na: 0	N: 0	Na: 2
**Gamma subband**	30–50 hz: 5	30–50 hz: 1	30–50 hz: 0	30–50 hz: 10
	30–70 hz: 5	30–70 hz: 0	30–70 hz: 0	30–70 hz: 2
	30–100 hz: 3	30–100 hz: 2	30–100 hz: 2	30–100 hz: 2
**Artifact correction method**	ICA: 0	ICA: 0	ICA: 0	ICA: 2
	ICA+other: 7	ICA+other: 0	ICA+other: 2	ICA+other: 6
	Other: 6	Other: 3	Other: 0	Other: 6
**Absolute or relative power**	Absolute: 9	Absolute: 2	Absolute: 2	Absolute: 8
	Relative: 1	Relative: 0	Relative: 0	Relative: 4
	Na: 4	Na: 1	Na: 0	Na: 2
**Sensor-level spectral power vs source estimates**	Sensor: 7	Sensor: 1	Sensor: 0	Sensor: 8
	Source: 6	Source: 2	Source: 2	Source: 5
				Both: 1
**Brain region**	Whole brain: 3	Whole brain: 0	Whole brain: 0	/
	Multiple: 7	Multiple: 2	Multiple: 2	
	Frontal: 1	Frontal: 0	Frontal: 1	
	Temporal: 1	Temporal: 1	Temporal: 0	
	Central: 1	Central: 0	Central: 0	
	Parietal: 0	Parietal: 0	Parietal: 0	
	Occipital:0	Occipital: 0	Occipital: 0	
**Source localization method**	LORETA: 2	LORETA: 1	LORETA: 0	LORETA: 2
	Equivalent current dipole: 3	Equivalent current dipole: 0	Equivalent current dipole: 0	Equivalent current dipole: 0
	Minimum norm estimates: 2	Minimum norm estimates: 0	Minimum norm estimates: 0	Minimum norm estimates: 1
	DICS: 0	DICS: 0	DICS: 2	DICS: 1
	BESA: 0	BESA: 0	BESA: 0	BESA: 2
	Others: 0	Others: 1	Others: 0	Others: 1

Local or whole brain spectral changes compared to healthy controls are considered. Values of numeric variables are expressed as median [p25 | p75]. For categorical variables, the number of studies is reported. *DICS* Dynamic Imaging of Coherent Sources, *BESA* brain electrical source analysis.

The study from Venables et al., 2009, and the one from Grent’-t-Jong et al., 2018, were considered twice, for eyes open and eyes closed condition and for first episode of psychosis and chronic schizophrenia, respectively.

^a^Cohort including both medicated and unmedicated patients.

**Table 6:** Clinical, preprocessing and analysis factors implicated in heterogeneity of findings for functional connectivity (patients vs controls).

Parameter	Studies with increased gamma functional connectivity (*N* = 4)	Studies with decreased gamma functional connectivity (*N* = 6)	Studies with mixed results (*N* = 4)	Studies with no group differences (*N* = 6)
**Total sample size**	147	179	168	260
**Age**	29 [27 | 31.3]	28.1 [27.3 | 40]	40.4 [37 | 40.4]	24 [23.5 | 36.7]
**Sex (F)**	32% [26 | 36.5]	48% [31 | 53]	21% [21 | 21]	24% [23 | 37]
**Duration of illness (months)**	63.2 [43.7 | 117.1]	24.2 [18.4 | 47.1]	12.7 [12.7 | 12.7]	19.3 [17.7 | 48.1]
**Severity of disease (PANSS total score)**	75.8 [70 | 78.2]	64.6 [61.4 | 72.2]	91 [91 | 91]	62.1 [58 | 68]
**Medication status**	Medicated: 1	Medicated:3	Medicated: 3	Medicated: 4
	Unmedicated: 2	Unmedicated: 0	Unmedicated: 0	Unmedicated: 0
	Mixed^a^: 1	Mixed^a^: 2	Mixed^a^: 0	Mixed^a^: 2
	Na: 0	Na: 1	Na: 1	Na: 0
**Recording method**	EEG: 4	EEG: 3	EEG: 4	EEG: 3
	MEG: 0	MEG: 2	MEG: 0	MEG: 3
**Number of channels/sensors**	114 [32 | 194]	45 [20 | 306]	64 [64 | 64]	25 [21 | 148]
**Recording duration**	5’ [3 | 8]	7’ [5 | 12]	4’ [4 | 5]	7’ [5 | 10]
**Eyes open/eyes closed**	Open: 0	Open: 2	Open: 1	Open: 1
	Closed: 4	Closed: 3	Closed: 3	Closed: 5
	Na: 0	Na: 1	Na: 0	Na: 0
**Gamma sub-band**	30–50 hz: 2	30–50 hz: 3	30–50 hz: 1	30–50 hz: 4
	30–70 hz: 1	30–70 hz: 2	30–70 hz: 3	30–70 hz: 2
	30–100 hz: 1	30–100 hz: 1	30–100 hz: 0	30–100 hz: 0
**Artifact correction method**	ICA: 1	ICA: 0	ICA: 0	ICA:
	ICA+other: 1	ICA+other: 1	ICA+other: 3	ICA+other:
	Other: 2	Other: 4	Other: 1	Other:
	Na: 0	Na: 1	Na: 0	Na:
**Absolute or relative power**	Absolute: 2	Absolute: 4	Absolute: 1	Absolute: 2
	Relative: 1	Relative: 1	Relative: 0	Relative: 1
	Na: 1	Na: 1	Na: 3	Na: 3
**Brain region**	Whole brain: 1	Whole brain: 3	Whole brain: 0	/
	Multiple: 3	Multiple: 3	Multiple: 4	
	Frontal: 0	Frontal: 0	Frontal: 0	
	Temporal: 0	Temporal: 0	Temporal: 0	
	Central: 0	Central: 0	Central: 0	
	Parietal: 0	Parietal: 0	Parietal: 0	
	Occipital: 0	Occipital: 0	Occipital: 0	
**Source localization method**	LORETA: 2	LORETA: 2	LORETA: 1	LORETA: 1
	Equivalent current dipole: 0	Equivalent current dipole: 0	Equivalent current dipole: 0	Equivalent current dipole: 0
	Minimum norm estimates: 0	Minimum norm estimates: 0	Minimum norm estimates: 0	Minimum norm estimates: 2
	DICS: 0	DICS: 0	DICS: 0	DICS: 0
	BESA: 0	BESA: 0	BESA: 0	BESA: 0
	Others: 0	Others: 1	Others: 0	Others: 1
**Sensor-level spectral power vs source estimates**	Sensor: 2	Sensor: 3	Sensor: 3	Sensor: 2
	Source: 2	Source: 3	Source: 0	Source: 4
	Both: 0	Both: 0	Both: 1	Both: 0
**Functional connectivity method:**	Phase lag index: 0	Phase lag index: 2	Phase lag index: 3	Phase lag index: 2
	Coherence: 1	Coherence: 2	Coherence: 0	Coherence: 0
	Envelope: 0	Envelope: 0	Envelope: 0	Envelope: 2
	Various: 0	Various: 1	Various: 1	Various: 0
	Others: 3	Others: 1	Others: 0	Others: 2

Local or whole brain spectral changes compared to healthy controls are considered. Values of numeric variables are expressed as median [p25 | p75]. For categorical variables, the number of studies is reported.

*DICS* Dynamic Imaging of Coherent Sources, *BESA* brain electrical source analysis.

Studies from Chang et al., 2024 (mixed results) and Alamian et al., 2020 (no difference) was considered 2 times, for eyes open and closed conditions;

^a^Cohort including both medicated and unmedicated patients.

The meta-analysis on the difference in gamma1 spectral power between patients and controls follows the other results on power spectra.

### Power spectra

Power spectra are the power distribution of EEG signal, decomposed in bands of frequency. The local electrical power is classically divided into 5 bands: delta (0–4 Hz), theta (4–8 Hz), alpha (8–12 Hz), beta (13–30 Hz) and gamma (30–100 Hz)^8^; this latter is further divided in gamma1 (30–49 Hz), gamma2 (50–70 Hz), gamma3 (71–100 Hz) and supergamma ( >100 Hz) subbands.

While the generation mechanisms at the cellular level do not change across the gamma spectrum, low-gamma and high-gamma oscillations are considered to have different functional specialization, with lower frequencies linked to attention and sensory processing, and higher gamma associated with more complex cognitive functions, such as language, memory and consciousness^11,42^.

#### Patients with schizophrenia vs healthy controls

Twelve studies indicated an increase in resting-state gamma power in schizophrenia^43–54^, 3 studies a reduction^55–57^, 1 study yielded mixed results (i.e., increase and decrease in spectral power in different brain regions), and 13 showed no difference in patients with schizophrenia vs healthy controls^58–70^.

Concerning studies having found an increase in gamma power in patients vs controls, the difference involved the whole brain (*N* = 3)^43–45^, multiple different regions (*N* = 6)^46,47,49,50,53,54^, or only the frontal (*N* = 1)^48^, central (*N* = 1)^52^ or temporal (*N* = 1)^51^ regions, in the left (*N* = 3)^48,51,52^, the right (*N* = 1)^57^ or both (*N* = 8)^43,45,46,49,50,53,54,71^ the hemispheres.

Significant differences between patients and controls were found in the gamma1 (*N* = 6)^43,46,52^, gamma1 and gamma2 (*N* = 3)^45,53,54^, or in the full gamma band (*N* = 2)^51,71^.

Concerning studies having found a decrease in gamma power in patients vs controls, the difference was localized in multiple regions (*N* = 2)^55,56^ or in the temporal lobe only (*N* = 1)^57^, involving the right hemisphere (*N* = 1)^57^ or both (*N* = 2)^55,56^, in the gamma1 (*N* = 1)^57^, gamma3 (*N* = 1)^55^ or the full gamma band (*N* = 1)^56^.

One study showed a pattern of increases and decreases of the whole-band gamma power, widespread to multiple regions in both hemispheres. This study also suggested that findings could depend on phase and duration of the disorder: in fact, compared to healthy controls, first episode of psychosis patients showed decreased prefrontal and increased occipital gamma1 activity, while chronic patients showed a decreased gamma1 in the frontal, temporal and sensorimotor areas^71^.

Studies detecting no differences between patients and controls in gamma spectral power analyzed specific regions (i.e., frontal, temporal, central, parietal and occipital) in both hemispheres, with few exceptions: one study focused on whole brain measures^60^, one study only considered regions belonging to the default mode network (i.e., posterior cingulate cortex, medial prefrontal cortex, lateral inferior parietal cortex, precuneus and anterior cingulate cortex)^62^, and two study focused on the auditory cortex^64,65^.

Gamma1 (*N* = 9)^66,69,70^, gamma1 and gamma2 (*N* = 2)^67,72^ or the whole band (*N* = 2)^64,65^ were analyzed.

Of the reviewed studies, only 5 used MEG^53,56,62,67,71^, while the others were conducted with an EEG, and several different methods were used for source localization: LORETA (low resolution brain electromagnetic tomography; *N* = 5^48,54,57,62,63^), dynamic imaging of coherence sources (DICS; *N* = 2^59,71^), BESA (brain electric source analysis; *N* = 2)^64,65^, equivalent current dipole (ECD) methods (*N* = 3)^43,49,51^, others (*N* = 4)^53,56,60,67^. These studies conducted the analysis at the source level, while the others at the sensor level, with 2 investigations using both^54,63^. Nine studies were conducted in eyes open conditions^43,49,50,59,62,67,70,71^, 1 study in both eyes open and closed^53^, the others (*N* = 22) with eyes closed. Further details on differences in recording, preprocessing and analysis are provided in the Table 5.

Another feature investigated was the relative amount of gamma power compared to the whole spectral power. In one investigation, patients’ activity was the greatest in gamma1, unlike in healthy controls, for whom activity was the greatest in alpha2^48^.

In one study the distribution of gamma power across the brain was different between patients and healthy controls in gamma1 and gamma2 subbands^73^, but no differences emerged in this respect in another study, focused on gamma1^46^.

In summary, the results were heterogeneous and do not allow to draw solid conclusions: while almost half of the studies detected no significant difference in gamma power between patients and controls, others suggested that schizophrenia is associated with altered gamma power. Twelve studies indicated an increased gamma activity in several regions and subbands, and only 3 observed a reduction.

#### Symptom severity

Several studies, already reported in the previous paragraph, have also assessed the relationship between symptom severity and resting state gamma-band power spectra, including positive symptoms, negative symptoms, and other dimensions, such as cognition and insight. Findings hereafter enumerated derive from single studies, without replication from one to the other.

Overall severity of disease positively correlated to gamma power either in the whole brain^71^, or in the prefrontal^62^ and the occipital cortex^46^; overall severity of disease also negatively correlated to gamma1 and gamma2 power in the temporal region^44^. Differences were lateralized to the left^44,46^ or to both^62,71^ the hemispheres. Subbands involved were gamma1 and gamma3^71^, gamma1 and gamma2^44^, or gamma1 only^46,62^. A negative correlation emerged between right temporal gamma3 power and social anhedonia^55^.

Gamma1 power significantly correlated with insight in left frontal and central regions^52^, but not with negative symptoms^52^.

Eight studies, whose methods are summarized in the previous paragraph and in Tables 1–3, revealed no significant relationship between the resting-state gamma-band and positive and negative psychotic symptoms severity^48,52,53,61,62,67,72,74^.

Concerning cognition, overall measures as the Brief Assessment of Cognition in Schizophrenia scale (BACS) and the Comprehensive Assessment of At-Risk Mental States negatively correlated with gamma power in posterior areas in patients with a first episode of schizophrenia, while frontal and central areas showed the opposite relationship. In chronic schizophrenia, BACS score was positively correlated with gamma-power, especially with gamma1^71^.

Other studies found no association of gamma power with working memory^49^ and with verbal learning^43^.

In summary, research provides heterogeneous and non-replicated findings, with both positive and negative correlations observed in different brain regions, across several gamma subband. Such results cast doubts on the existence of a sound relationship between gamma-related spectral features and symptoms of schizophrenia.

#### Other results

Gamma-power negatively correlated with the duration of illness in the bilateral frontal regions in the gamma1 and gamma3 bands and in the right temporal region for gamma3 only^45^.

One investigation observed an increase in whole-brain gamma1 power in bipolar disorder compared to schizophrenia^60^.

### Gamma1 power spectra meta-analysis

**Fig. 2:**
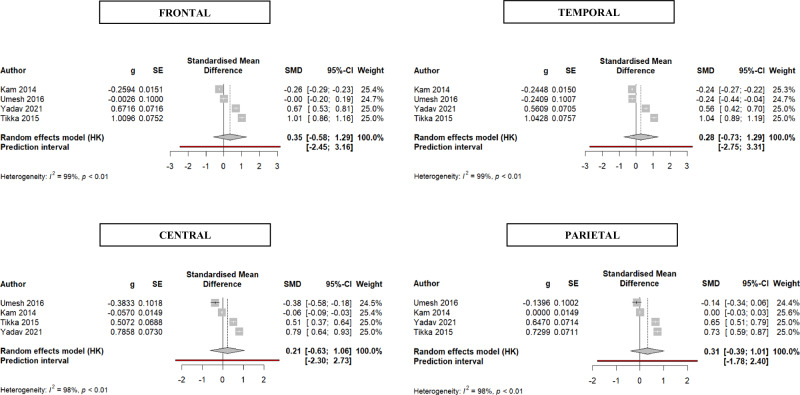
Forest plot of standardized effect sizes (Hedge’s g) of gamma1 power in patients with schizophrenia vs healthy controls.

All the records were initially included for meta-analyses on power spectra or functional connectivity differences between patients and controls. Only 19 studies provided exhaustive quantitative data in the text and in Supplementary Materials. Parameters for whom at least 3 studies had a comparable method (i.e., power spectra values in the whole brain or in single regions; specific measures of functional connectivity) were kept. Following this procedure only papers focused on power spectra remained (*N* = 7), but they were inhomogeneous for the frequency subband analyzed and for the use of absolute or relative power. In the end, the meta-analyses was conducted on studies providing measures of absolute power in the gamma1 band (*N* = 4), in specific brain regions (i.e., frontal, central, parietal, temporal). Individual study (*k* = 4) and aggregate effect sizes and measures of heterogeneity are presented in detail in Fig. 2.

Gamma1 power was not significantly increased in patients vs controls, in any region under study. Although all effect sizes were positive (i.e., higher gamma in patients vs controls), they were small and had broad confidence intervals. No outliers were detected.

An influence analysis with a leave-one-out method did not allow for a significant reduction of heterogeneity. The low number of studies included in the meta-analysis (*n* = 4) prevented subgroup analysis, meta regression, and the assessment of publication bias^39^.

### Functional connectivity

EEG functional connectivity is the temporal coincidence of spatially distant neural activities that likely reflects the dynamic interregional communications in the brain, and it is calculated for all the frequency bands^8^.

#### Patients with schizophrenia vs healthy controls

Four studies observed an increase^44,63,74,75^, 6 studies a reduction^51,57,62,73,76,77^, 3 mixed results^54,76,78^ and 5 no difference between patients and controls^53,60,63,66,79^. Table 4 provides details on the localization and extension of the seed regions for the connectivity analysis, and on the regions with whom functional connectivity was reduced or increased.

An increased gamma-band connectivity was observed in the whole brain (*N* = 3)^44,74,78^ or involving multiple regions (*N* = 3)^63,75,76^. Differences were lateralized to the right in one case^75^, and involving both the hemispheres in the others.

A reduction in functional connectivity emerged in the whole-brain (*N* = 3)^73,76,77^, in multiple bilateral brain regions (*N* = 2)^51,62^, or in the right temporal and parietal regions (*N* = 1)^57^. Gamma subbands involved were gamma1 (*N* = 3)^57,62,76^, gamma1 and gamma2 (*N* = 2)^73,77^, or the full band (*N* = 1)^51^.

Three studies observed a complex pattern of decrease and increase in gamma connectivity across multiple bilateral brain regions, involving the gamma1 (*N* = 1)^76^, or gamma1 and gamma2 bands (*N* = 2)^54,78^. One study analyzing the functional connectivity at the whole brain level using multiple measures at the same time showed a complex pattern of increases or decreases in the various indexes considered^54^.

Four studies reported no significant differences in gamma band functional connectivity between patients and controls across all the brain regions, involving gamma1 (*N* = 3)^60,79,80^ or gamma1 and gamma2 bands (*N* = 1)^53^.

Differences in connectivity could also depend on the duration of disease, as suggested by one study reporting that patients with a more recent disease onset (< 5 years) had a diffuse increase in gamma1 and gamma2 connectivity compared to more chronic patients^75^. However, another study found no differences in the gamma1 connectivity between first-episode patients and chronic patients^66^.

Only 4 studies used MEG^53,62,77,79^, while the remaining 12 were conducted with EEG. For source localization, 7 studies used LORETA^54,57,62,63,75,76,80^, 2 minimum norm estimates (MNE)^53,77^ and two other methods^51,79^. While the studies enumerated conducted the analysis at the source level, the others were realized at the sensor level, with three exceptions using both^54,63,80^. Various methods were adopted for functional connectivity analysis, with 4 studies^66,73,76,78^ using the phase lag index (i.e., a bivariate measure of functional connectivity that and can be defined in terms of the Hilbert transformation of bandpass filtered signals, or in terms of the time-frequency representation of the broadband data^81^), 4 studies^44,51,60,62^ using measures of coherence (i.e., the consistency of the relative amplitude and phase between signals detected in coils or electrodes within a set frequency band^82^), 2 studies^63,79^ power envelope correlates (i.e., the relationship between the amplitude of the oscillatory brain activity and the temporal dynamics of that activity across different regions of the brain^83^), one study integrating several different analysis methods^54^ and 5 studies using other techniques^53,57,74,75,80^. In two cases the recording was with eyes open^62,77^, in other two with both eyes open and closed^53,78^, and with eyes closed only in the remaining studies (*N* = 14). Further details on differences in recording, preprocessing and analysis are provided in the Table 6.

In summary, the findings regarding gamma-band functional connectivity in schizophrenia are mixed and not conclusive, in some case partly overlapping from one study to the other, but without a consistent replication. Both increase and decrease in functional connectivity were described within and across brain areas, involving the three gamma subbands.

#### Symptom severity

Concerning positive psychotic symptoms, 3 studies produced disparate evidences.

Patients with more severe disorganization, delusions and hallucinations showed a lower gamma1 connectivity in a network including the left rolandic operculum, the left temporal regions and bilateral parietal, lateral frontal and orbitofrontal cortex^63^. In contrast, gamma1 and gamma2 whole brain connectivity was greater in patients having auditory verbal hallucinations than in controls^84^. Last, mixed results emerged in a study showing a positive correlation of PANSS total score with gamma1 and gamma2 local efficiency (i.e., he local information transmission capability of the network) in the frontal lobe and a negative correlation of the PANSS negative score with node degree (i.e., the number of edges connected to a node in a graph)^78^.

Attention had a significant positive association with gamma1 connectivity between the right cuneus and two regions: the transverse temporal gyrus and the superior temporal gyrus^85^.

No differences emerged in other investigations (*N* = 6) when considering the associations of gamma1 connectivity with severity of psychotic symptoms^54,74^, cognitive processing speed^76^, performance initiation^57^, and performance on arithmetic tasks^61,76^.

In summary, the literature provides only hints to a link between severity of schizophrenia symptoms and functional connectivity alterations, without replicated findings.

### Phase amplitude coupling

A core mechanism of brain functioning is that low-frequency characteristics such as phase, amplitude and power, influence and synchronize those of higher frequency, an effect called phase-amplitude coupling^86^. This mechanism underlies the brain’s ability to integrate information across different frequency bands, allowing for coordinated processing among neural networks^87^.

Patients had higher values of theta-gamma coupling than controls in the whole brain^88^, in bilateral prefrontal areas^89^ or in the posterior cingulate cortex^90^. A study focused on the auditory cortex found no significant differences between patients and controls concerning theta-gamma coupling, that was however lateralized to the left in healthy subjects, but not in patients with schizophrenia^65^.

Moreover, theta-gamma coupling values in the posterior cingulate cortex were correlated with the results of the trail-making A, trail-making B, immediate recall and delayed recall tests^90^.

Delta-band phase synchronization with high gamma power^91^ was decreased in patients compared to healthy subjects in a wide region including bilateral frontocentral and temporoparietal electrodes.

Alpha-gamma coupling was greater in controls than in patients, in the whole brain^89^.

In summary, theta-gamma coupling was more often increased in patients with schizophrenia compared to healthy controls, at the whole brain or at the local level, with hints to a relationship with cognitive functioning. Other forms of phase amplitude coupling were rarely studied.

## Discussion

Overall, our systematic review of 2133 patients and 2040 controls shows the heterogeneity of the existing literature on resting-state gamma frequency in patients with schizophrenia, concerning both methods and results. Although several studies have suggested an increased power and disrupted connectivity of resting-state gamma frequencies in schizophrenia, findings were not consistent across investigations, and not supported by our preliminary meta-analysis. The overall picture is inconsistent, with complex patterns of increased and decreased gamma power and connectivity. These patterns were either extended to the whole brain or located in specific regions. The reproducibility of the results from one study to the other was low, also partly due to the use of different methods for EEG/MEG analysis and brain area segmentation.

### Power spectra

Twelve studies reported increased gamma-power in patients versus controls. Even with a significant heterogeneity concerning lateralization and region, the difference was widespread to most brain regions, more often involving multiple areas at the same time, and more consistently localized in the bilateral frontal and temporal cortex. However, three studies revealed reduced gamma-power in key regions such as prefrontal cortex, precuneus, cuneus, cingulate gyrus and posterior cingulate cortex and postcentral gyrus^55–57^. Finally, 13 studies did not find differences between patients and controls. These findings mostly concerned the gamma1 band, with a fewer reports on gamma2 and gamma3. We were only able to include 4 studies in an exploratory meta-analysis, that did not support the hypothesis of an increased gamma1 power in schizophrenia. Only minor differences between patients and controls emerged in frontal, temporal, parietal and central areas, not leading to statistical significance and with heterogeneity values of 98–99%.

One study^71^ suggested that gamma-band features could change according to the phase and duration of the disease. The severity of the disease correlated with gamma activity in the whole brain^71^, or in the frontal^62^, temporal^44^, and occipital^46^ regions, however 8 studies found no associations in this respect.

Concerning cognition, one study gave initial hints of a relationship between overall functioning and the gamma activity in frontal and occipital regions^71^, but 2 other investigations led to non-significant results.

The inconclusive findings on resting-state gamma frequencies in patients with schizophrenia are surprising, because in contrast with a large body of coherent preclinical evidences, indicating that an increase in gamma power (related to a glutamatergic dysfunction) leads to schizophrenia-like symptoms in both animal models and healthy volunteers. In fact, an increase in the excitatory input to parvalbumin interneurons in rodents via selective optogenetic stimulation leads to an increase in gamma activity, and the inhibition of parvalbumin interneurons leads to an immediate gamma suppression^92,93^. Moreover, the baseline gamma band in animal models of schizophrenia is elevated^94^. In rodents, pyramidal cell excitability and spontaneous gamma power are increased by NMDA-R antagonists^95–98^ and by a genetic loss-of-function of NMDA-R subunits^28–30^ or of functionally related genes such as ErbB2^31^. Additionally, mutant mice expressing 5–10% of the obligatory NMDAR1 subunit exhibited electrophysiological and behavioral deficits consistent with schizophrenia^32–34^. A consistently replicated postmortem finding in the cortex of individuals with schizophrenia is the reduced expression of mRNA encoding the 67 kD isoform of GAD^35^. The use of NMDA-R antagonists (e.g., ketamine, PCP and MK-801) was associated with increased auditory-evoked gamma oscillation^99^, motor cortex excitability, psychotic symptoms and cognitive impairment in healthy volunteers^27^. Last, limbic encephalitis due the autoimmune antibodies binding to NMDA receptor can present with symptoms of schizophrenia, fostering the role of NMDA in the pathogenesis of this disorder^100^.

### Functional connectivity and phase amplitude coupling

Gamma oscillatory activity is thought to be a fundamental mechanism that integrates neural networks within and across brain structures, facilitating coherent sensory elaboration and cognitive operations^101^. In particular, gamma frequencies are the key for feature binding (i.e., the ability to integrate different sensory features of a stimulus into a single coherent neural representation^102^).

In the studies included in this review, several different measures were used, leading to inconsistent findings. The few studies reporting significant effects suggest a complex global picture, with patterns of a decreased and/or increased functional connectivity across the whole brain, involving multiple different brain regions in each study. Disease severity correlated with frontal^63^ or whole brain^84^ gamma connectivity, but with contradictory results in another study^78^, and no significant associations in the others. Concerning cognition, only one study observed an association between attention and temporal lobe connectivity^85^.

Phase amplitude coupling was the focus of several investigations, with some studies suggesting increased theta-gamma coupling in patients with schizophrenia^88–90^, while in single studies delta-band phase synchronization with high-gamma was decreased^91^ and alpha-gamma coupling was increased^89^ in patients compared to healthy controls.

Evidence on the abnormal coordination of gamma oscillations in schizophrenia^103,104^ points toward a disconnection hypothesis, characterized by an aberrant synaptic modulation both between brain regions and between the layers of cortical columns^105,106^, leading to the disruption of networks underlying cognition and emotional processing^107,108^. Even if low-frequency oscillations impose fewer constraints on the precision of timing^109^ and are therefore probably more relevant for long-range synchronization, also gamma plays a role in this respect^11,110^.

### Resting-state and task related gamma frequencies in schizophrenia

While our review of resting state gamma oscillation does not yield consistent effects, findings on task-related gramma responses seem to be more conclusive, as indicated by recent systematic reviews. Task-related responses in the gamma range were investigated especially in the auditory modality, with only one study dealing with the visual evoked response, which was reduced in schizophrenia^111^. The 40 Hz auditory steady state response (ASSR) in schizophrenia has deficient intertrial phase coherence and power, and is phase delayed compared to the norm; moreover, auditory steady-state response propagation across cortical sources was aberrant^25^. The consistent pattern for the ASSR indicates that circuits involved in the generation of high frequency oscillations are compromised; the inconclusive findings on resting state gamma suggest that the impairment involves mechanisms that are specific for task induced oscillations, not involving the resting-state.

Preclinical studies based on the administration of ketamine held the potential to better ascertain the link of the NMDA receptor activity with changes in gamma band oscillations. While in the resting state an increased gamma power after ketamine administration was observed in most of the studies^26^ results on task related recordings were mixed. In healthy controls, studies reported an increased gamma during an auditory paired click task^112^ and during a visual motor task^113^, but also a reduction during an auditory evoked gamma band response task^114^. During a visual grating task, ketamine induced an increase in gamma activity in healthy controls and a reduction in participants with schizophrenia^115^. These contradictory results indicate a complex dynamic between ketamine, NMDA receptor activity, and gamma band oscillations.

### The translational potential of resting-state gamma frequencies

Resting-state gamma frequencies hold the potential for being a translational biomarker. On one hand, they could be useful for the diagnosis of schizophrenia and the assessment of its severity; however, heterogeneity of findings, as discussed, hinders such an application. On the other hand, gamma frequencies configure as a neurophysiology features to target with therapeutic interventions, such as medications and non-invasive brain stimulation.

Several approaches were developed to pharmacologically correct the activity of the NMDA receptor; the major challenge in this respect being that, while NMDA receptors are widespread to the whole brain, the pathophysiology of schizophrenia may be confined to specific brain regions and to discrete cell types, such as the PV-interneurons. Moreover, potentiating the glutamatergic synaptic activity may lead to excitotoxicity^22^. For these reasons, several compounds were developed to augment NMDA signaling indirectly, by enhancing glycinergic neurotransmission. Even if initial evidence of an efficacy on positive, negative, and cognitive symptoms emerged, larger clinical trials and meta-analyses revealed a modest overall effect^22,116^.

An opposite approach consists in antagonizing the activity of the NMDA receptor: riluzole showed a modest potential for treating negative symptoms^117,118^, while lamotrigine had a limited efficacy as an adjunctive medication for clozapine-resistant schizophrenia^119^. Enhancing the activity of the alpha-2 subunit containing GABA-A receptor leads to a reduced downstream glutamate release: a partial agonist at alpha 2 and alpha 3 GABA-A receptor subunits (MK-0777) improved cognition in patients with schizophrenia, but no effect on positive and negative symptoms was observed^120^.

Several studies showed an effect of antipsychotic medications on resting state gamma oscillations, and some of these related to response to treatment^47,121,122^, making it worth to further study their predictive power on response to medications.

The limited effectiveness of pharmacological interventions targeting glutamate prompts clinical trials on different therapeutic approaches, especially the ones based on non-invasive brain stimulation.

Transcranial magnetic stimulation proved to be effective in the treatment of positive and negative symptoms of schizophrenia, especially when applied to the dorsolateral prefrontal and the temporal cortex^123,124^.

Transcranial magnetic stimulation and direct current stimulation can entrain brain oscillations and modulate brain areas in a frequency-dependent and long-lasting manner^125^, and this effect seems to be mostly mediated by GABAergic interneurons activity^11,126^. TMS demonstrated to interact with gamma frequencies^127–129^, and a pilot study indicates that a modification of gamma frequencies by mean of TMS correlates with an improvement of disrupting behaviors in patients with autism^130^. However, a similar investigation focused on working memory in schizophrenia led to no significant results^131^. Further clinical trials are warranted to assess if an intervention targeting the resting-state gamma to make their pattern closer to the one of healthy controls can be an effective treatment of schizophrenia.

### Limitations

Our systematic review and meta-analysis was limited by the methodological heterogeneity of included studies which does not allow to draw univocal and generalizable conclusions. Clinical and demographic variables and recording, preprocessing and analysis methods of the EEG/MEG data were considered as potential bias on results, possibly explaining the different and sometimes contradictory outcomes of different studies.

Intersubjective variability in sociodemographic and clinical features such age, sex, educational level, duration, and severity of disease was considerable and these parameters have been demonstrated to influence EEG in schizophrenia^132^. Some studies did not report relevant data about sociodemographic and clinical characteristics, and patients were studied in different phases, concerning duration of disease and inpatient/outpatient status. Since EEG features are likely to change along the course of the disorder, this inhomogeneity represents a major limitation^71^. Patients were treated with different antipsychotic medications: the effect of each one on EEG trace can be specific and the responder vs non-responder to treatment status is associated with different EEG patterns^133^, leading to further heterogeneity. Many important pieces of information about medication were often missing, such as the dose, duration of treatment, concurrent and previous medications; when these informations were offered, large variability was observed among studies and subjects under study.

In the eyes-closed condition, there is a notable change in the dynamics of gamma band activity, which typically decreases, while alpha activity increases; this shift in frequency band activity can potentially drive a difference in results of studies conducted in the eyes open or eyes closed condition, which could be relevant given that the proportion of studies in the two conditions was not equal (eyes open: *N* = 11; eyes closed: *N* = 30; both: *N* = 3).

In some of the studies the duration of recording could be considered as suboptimal^46,61,67^.

The choice of source localization methods significantly influences EEG/MEG results due to varying precision and sensitivity to artifacts. Techniques such as Synthetic Aperture Magnetometry (SAM) and DICS excel in spatial resolution and network connectivity insights, respectively; however, both can be affected by noise. eLORETA and sLORETA provide stable localization at the cost of spatial specificity, while the Current Source Density (CSD) method effectively captures local activity but is sensitive to reference choices and heavily rely on an accurate head model. The ECD model may oversimplify complex activity patterns, and methods like MNE can struggle in underdetermined scenarios^134^.

Moreover, brain segmentation and techniques for attributing activity to sources differed by study, making it hard to exactly compare topographies, even when findings were in overlapping regions.

The intra- and intersubject variability of EEG/MEG recordings was another major concern: studies are generally characterized by a high variability within and even more between subjects, which might not always be indicative of any pathological status, making it difficult to generalize reports without applied normalization.

Functional connectivity techniques were different and non-comparable among different studies. Moreover, features as coherence, correlation and mutual information are highly subject to the volume conduction effect (i.e., at the level of each EEG channel, the effect of a mixture of active brain and nonbrain electrical sources whose activities are conducted to the scalp), which could produce results incorrectly indicating a functional connectivity or hemispheric asymmetries^135^. While we do not anticipate volume conduction to affect patients and controls differently, its impact could lead to more heterogeneous and less precise results, increasing uncertainty and reducing comparability between groups.

Gamma frequencies are subjected to contamination by muscular sources, especially in the gamma2 and gamma3 subbands^8^; for example, the myogenic activity from oculomotor and cranium foramen muscles generates biphasic sharp potential transients at the onset of a saccade, generating a broadband power increase in the 20–200 Hz range^136,137^.

This systematic review and meta-analysis present a significant risk of publication bias, which however was mitigated by the fact that several studies were not focused on resting-state gamma band, but also on other outcomes, which led to positive findings and, as a consequence, more likely to publication.

The small number of studies included in the meta-analysis precludes the generalizability of findings; only few were sufficiently homogeneous to conduct a meta-analysis, and potentially included data were not available for some studies. In addition, by including only studies published in peer-reviewed journals it is possible that some data available in the literature may have been excluded (i.e., gray literature, non-English language). According to the Cochrane Handbook for Systematic Reviews^138^, the low number of studies hindered second-level analysis useful to individuate bias and to moderate their effect, such as meta-regression and subgroup analysis. Also, publication bias was not assessed with a rigorous method (e.g., Egger’s linear regression^139^). In the end, this meta-analysis should be considered as purely exploratory.

When evaluating the relationship to positive and negative symptoms, studies included often used the PANSS positive and negative subscales, an approach that is now considered outdated; a more meaningful analysis would imply the use of the five factors model^140^.

Last, while EEG and MEG activity have been predominantly interpreted in the terms of the distribution of frequency along power spectra, as reflected in the present review, recent time have seen a renewed interest in the non-oscillatory, aperiodic component of the signal, as a unitary measure accounting for brain spectral power across frequencies^141^. The change in gamma band power in schizophrenia could manifest as a change in the aperiodic component, especially when accompanied by an inverse change in lower frequency bands. Only few studies to date evaluated the aperiodic component in schizophrenia, with only two studies in the resting state^142^, a number unsuitable for a systematic review. More importantly, the concept of aperiodic component involves gamma band only as a part of a broader phenomenon, and does not represent a gamma-band parameter in itself. For these reasons, we decided to not to include this aspect in the present paper.

## Conclusions

Resting gamma-frequencies in schizophrenia were studied in a number of studies, leading to heterogeneous results. In our opinion, further investigations are needed. On one side they should be enlightened by novel theories on brain functioning and organization are needed for a unitary interpretation of the findings; on the other side, a standardization of methods and reporting would be necessary for a better interpretation of brain activity, and to determine its relevance for schizophrenia pathophysiology, diagnosis and treatment. Moreover, the heterogeneity of findings could be “real”, depending on the existence of different subgroups of patients with schizophrenia, having a distinctive pathophysiology and, accordingly, different EEG and MEG features. Schizophrenia is a heterogenous disorder, and the glutamatergic dysfunction underlying the dysregulation of gamma frequencies in some individuals may not be relevant, or may play a role only as one of several pathological mechanisms^22^.

Finally, few studies have evaluated the relationship between gamma frequency and symptoms, including the positive, negative and cognitive dimensions; this sparseness of the literature further prompts future investigations.

## Supplementary information

supplementary materials

## Data Availability

The data used in this systematic review and meta-analysis are sourced from the published studies revised. All relevant data supporting the findings of this study are included within the manuscript and its Supplementary Materials. Specific datasets analyzed during the study can be obtained from the respective articles referenced in the review. No original raw data were generated during this research, as it synthesized existing literature. Data files and the R script for the meta-analysis can be found on the open science framework (OSF).
