# Author Correction: Resting-state EEG and MEG gamma frequencies in schizophrenia: a systematic review and exploratory power-spectrum meta-analysis

**DOI:** 10.1038/s41537-025-00611-3

**Published:** 2025-04-12

**Authors:** Marco De Pieri, Michel Sabe, Vincent Rochas, Greta Poglia, Javier Bartolomei, Matthias Kirschner, Stefan Kaiser

**Affiliations:** 1 Division of Adult Psychiatry, Department of Psychiatry, University Hospitals of Geneva, Thonex, Switzerland; 2 Faculty of Medicine, University of Geneva, Geneva, Switzerland; 3 Functional Brain Mapping Laboratory, Department of Basic Neurosciences, University of Geneva, Geneva, Switzerland

**Keywords:** Biomarkers, Neural circuits

Correction to: *Schizophrenia* 10.1038/s41537-025-00596-z, published online 21 March 2025

In this article, “Metanalysis” was incorrectly used in the title and throughout the manuscript. It should have been “meta-analysis.” The original article has been corrected.

