# Supplementary material for: Resting-state EEG and MEG gamma frequencies in schizophrenia: a systematic review and exploratory power-spectrum meta-analysis
Source: Schizophrenia (Heidelb). 2025 Mar 21;11(1):48. doi: 10.1038/s41537-025-00596-z (PMC11933325; doi:10.1038/s41537-025-00596-z)
Supplement: Supplementary file 1 — supplementary materials [file 41537_2025_596_MOESM1_ESM.docx]

**SUPPLEMENTARY MATERIALS**

**Supplementary table S1**: Newcastle Ottawa scale for the assessment of bias in case-control studies

|  |  | Selection | | | | Comparability | | Exposure | | Total | |
| --- | --- | --- | --- | --- | --- | --- | --- | --- | --- | --- | --- |
|  | Adequate case definition | Representativeness of the exposed cohort | Selection of controls | Definition of controls |  | | Same methods of ascertainment for cases and controls | |  | |  |
| *Rutter*  *2009* | * | * | * | * | * | | * | | 6 | |  |
| *Venables*  *2009* | * | * | * | * | ** | | * | | 7 | |  |
| *Kikuchi*  *2011* | * | * |  | * | * | | * | | 5 | |  |
| *Hanslmayr*  *2012* | * | * |  | * |  | | * | | 4 | |  |
| *Andreou*  *2014a* | * | * | * | * | ** | | * | | 7 | |  |
| *Andreou*  *2014b* | * | * | * | * | ** | | * | | 7 | |  |
| *Garakh*  *2014* | * | * |  | * | ** | | * | | 6 | |  |
| *Kam*  *2014* | * | * | * | * | * | | * | | 6 | |  |
| *Kim*  *2014* | * | * | * | * | ** | | * | | 7 | |  |
| *Di Lorenzo*  *2015* | * | * |  | * | ** | | * | | 6 | |  |
| *Hirano*  *2015* | * | * | * | * | ** | | * | | 7 | |  |
| *Mitra*  *2015* | * | * |  | * |  | | * | | 4 | |  |
| *Tikka*  *2015* | * | * | * | * | ** | | * | | 7 | |  |
| *Ramyaed*  *2016* | * | * |  | * |  | | * | | 4 | |  |
| *Umesh*  *2016* | * | * |  | * |  | | * | | 4 | |  |
| *Won*  *2017* | * | * | * | * | * | | * | | 6 | |  |
| *Arikan*  *2018* | * | * |  | * |  | | * | | 4 | |  |
| *Baradits*  *2018* | * | * | * | * | ** | | * | | 7 | |  |
| *Grent-t’-Jong*  *2018* | * | * | * | * | ** | | * | | 7 | |  |
| *Hirano*  *2018* | * | * | * | * | ** | | * | | 7 | |  |
| *Jonak*  *2018* | * | * | Na | * | Na | | * | | 4 | |  |
| *Krukow*  *2018* | * | * | * | * | ** | | * | | 7 | |  |
| *Takahashi*  *2018* | * | * |  | * | * | | * | | 5 | |  |
| *Zeev-Wolf*  *2018* | * | * | * | * | ** | | * | | 7 | |  |
| *Lottman*  *2019* | * | * |  | * | ** | | * | | 6 | |  |
| *Vignapiano*  *2019* | * | * | * | * | ** | | * | | 7 | |  |
| *Alamian*  *2020* | * | * | * | * | * | | * | | 6 | |  |
| *Freche*  *2020* | * | * | * | * | * | | * | | 6 | |  |
| *Kim*  *2020* | * | * | * | * | ** | | * | | 7 | |  |
| *Krukow*  *2020* | * | * | * | * | ** | | * | | 7 | |  |
| *Lee*  *2020* | * | * |  | * | * | | * | | 7 | |  |
| *Soni*  *2020* | * | * |  | * | * | | * | | 5 | |  |
| *Tanaka-Koshiyama*  *2020* | * | * |  | * | * | | * | | 5 | |  |
| *Koshiyama*  *2021a* | * | * | * | * | * | | * | | 6 | |  |
| *Koshiyama*  *2021b* | * | * | * | * | * | | * | | 6 | |  |
| *Sun*  *2021* | * | * |  | * | * | | * | | 5 | |  |
| *Yadav*  *2021* | * | * | * | * | ** | | * | | 7 | |  |
| *Gordillo*  *2022* | * | * |  | * | * | | * | | 5 | |  |
| *Tagawa*  *2022* | * | * | * | * | * | | * | | 6 | |  |
| *Ibanez-Molina*  *2023* | * | * | * | * | ** | | * | | 7 | |  |
| *Jacob*  *2023* | * | * | * | * | ** | | * | | 7 | |  |
| *Yeh*  *2023* | * | * | Na | * | Na | | * | | 4 | |  |
| *Chang*  *2024* | * | * | * | * | * | | * | | 6 | |  |

In the exposure section the items “ascertainment of exposure items” and “non-response rate” were not included because not appropriate for the studies under review. Na not applicable. Scoring of studies follows this general principle 1-4 stars:  potential limitations in study quality, suggesting a higher risk of bias; 5-6 stars:  moderate quality, with some potential biases or limitations; 7-9 stars:  strong methodological quality, with minimal risk of bias. A maximum of 1 star can be attributed to each item, with the exception of comparability, that can receive 2 stars. A proportional scored was created for this review, given the number of not applicable items.

**Supplementary table S2**: Summary of studies having found positive results for regional differences in spectral power, functional connectivity and phase gamma coupling in patients compared to controls, and related to severity of psychotic or cognitive symptoms. Whole brain results are not reported.

|  | Spectral power | | Functional connectivity | | Phase amplitude coupling | | |
| --- | --- | --- | --- | --- | --- | --- | --- |
|  | Increased | decreased | Increased | Decreased | | Increased | Decreased |
| Patients vs controls | | | | | | | |
| Left frontal | (Freche et al., 2020; Mitra et al., 2015; Ramyead et al., 2016) | / | (Kikuchi et al., 2011; Tikka et al., 2015) | (Chang et al., 2024; Kim et al., 2014) | | (Ibáñez-Molina et al., 2023) | (Koshiyama et al., 2022) |
| Right frontal | (Freche et al., 2020; Koshiyama et al., 2021; Mitra et al., 2015) | / | / | (Chang et al., 2024; Kim et al., 2014; Krukow et al., 2018, 2020) | | (Ibáñez-Molina et al., 2023) | / |
| Left temporal | (Koshiyama et al., 2021; Mitra et al., 2015; Soni et al., 2020) | / | (Andreou et al., 2015; Chang et al., 2024; Takahashi et al., 2018; Tikka et al., 2015) | (Krukow et al., 2018; Soni et al., 2020) | | / | (Koshiyama et al., 2022) |
| Right temporal | (Mitra et al., 2015) | (Umesh et al., 2018) | (Chang et al., 2024; Krukow et al., 2018; Tikka et al., 2015) | (Krukow et al., 2018) | | / | (Koshiyama et al., 2022) |
| Left parietal | (Mitra et al., 2015) | (Rutter et al., 2009; Umesh et al., 2018) | / | (Soni et al., 2020) | | (Lee et al., 2020) | (Koshiyama et al., 2022) |
| Right parietal | (Mitra et al., 2015) | (Rutter et al., 2009) | / | (Kim et al., 2014; Soni et al., 2020) | | (Lee et al., 2020) | / |
| Central | (Arikan et al., 2018; Freche et al., 2020; Mitra et al., 2015) | (Umesh et al., 2018) | / | N= | | / | / |
| Left occipital | / | (Rutter et al., 2009; Umesh et al., 2018) | / | / | | / | / |
| Right occipital | / | (Rutter et al., 2009; Umesh et al., 2018) | (Di Lorenzo et al., 2015; Tikka et al., 2015) | / | | / | / |
| Severity of cognitive or psychotic symptoms | | | | | | | |
| Left frontal | (Arikan et al., 2018; Grent-’t-Jong et al., 2018; Kim et al., 2014) | / | (Chang et al., 2024) | (Andreou et al., 2015) | | / | / |
| Right frontal | (Grent-’t-Jong et al., 2018; Kim et al., 2014) | / | / | (Andreou et al., 2015) | | / | / |
| Left temporal | / | (Tikka et al., 2015) | / | (Andreou et al., 2015) | | / | / |
| Right temporal | / | (Tikka et al., 2015; Umesh et al., 2018) | / | / | | / | / |
| Left parietal | / | / | / | (Andreou et al., 2015) | | (Lee et al., 2020) | / |
| Right parietal | / | / | (Yeh et al., 2023) | (Andreou et al., 2015) | | (Lee et al., 2020) | / |
| Central | (Arikan et al., 2018; Grent-’t-Jong et al., 2018) | / | / | / | | / | / |
| Left occipital | (Baradits et al., 2019) | (Grent-’t-Jong et al., 2018) | / | / | | / | / |
| Right occipital | / | (Grent-’t-Jong et al., 2018) | / | / | | / | / |

**Supplementary table S3**: synopsis of clinical heterogeneity

| MEAN SEVERITY OF THE DISEASE (PANSS score) | | | | | | | | | |
| --- | --- | --- | --- | --- | --- | --- | --- | --- | --- |
| *30-50*  *pts** | *51-70*  *pts** | *71-90*  *pts** | *91-110*  *pts** | *>110 pts** | *Na* | | |  |  |
| Umesh et al., 2016 ; Zeev-Wolf et al., 2018 ; Lottman et al., 2019^#^  (N=3) | Andreou et al., 2014a; Andreou et al., 2014b; Kim et al., 2014 ; Ramyaed et al., 2016 ; Grent’t’ Jong et al., 2018 ; Jonak et al., 2018 (FEP cohort) ; Krukow et al., 2018 ; Kim et al., 2020 ; Krukow et al., 2020 ; Lee et al., 2020 ; Sun et al., 2021 ; Tagawa et al., 2022 ; Jacob et al., 2023 (N=13) | Venables et al., 2009^#^; Garakh et al., 2014 ; Kam et al., 2014 ; Di Lorenzo et al., 2015 ; Baradits et al., 2018 ; Jonak et al., 2018 (chronic cohort) ; Krishna-Tikka et al., 2020 ; Yeh et al., 2023  (N=8) | Kikuchi et al., 2011^#^ ; Mitra et al., 2015 ; Tikka et al., 2015 ; Takahashi et al., 2018^#^ ; Chang et al., 2024  (N=5) | N=0 | Rutter et al., 2009; Hanslmayr et al, 2012 ; Hirano et al., 2015° ; Won et al., 2017 ; Arikan et al., 2018° ; Hirano et al., 2018° ; Vignapiano et al., 2019° ; Alamian et al., 2020° ; Freche et al., 2020° ; Soni et al., 2020 ; Tanaka-Koshiyama et al., 2020 ; Koshiyama et al., 2021a ; Koshiyama et al., 2021b° ; Park et al., 2021 ; Gordillo et al., 2022 ; Ibanez-Molina et al., 2023; Chen et al., 2024 (N=17) | | |  |  |
| MEAN AGE (years) | | | | | | | | | |
| *<20* | *20-30* | *31-40* | *41-50* | *>50* | *Na* | | |  |  |
| N=0 | Andreou et al., 2014a; Andreou et al., 2014b; Garakh et al., 2015; Grent-’t-Jong et al., 2018; Hanslmayr et al., 2012; Jonak et al., 2019; Kikuchi et al., 2011; J. S. Kim et al., 2014; Krukow et al., 2018, 2020; Lee et al., 2020; Lottman et al., 2019; Mitra et al., 2015; Najafzadeh et al., 2021; Ramyead et al., 2016; Soni et al., 2020; Takahashi et al., 2018; S. K. Tikka et al., 2015; Umesh et al., 2018 (N=19) | (Arikan et al., 2018; Baradits et al., 2019; Chen et al., 2024; Di Lorenzo et al., 2015; Freche et al., 2020; Gordillo et al., 2023; Grent-’t-Jong et al., 2018; Ibáñez-Molina et al., 2023; Jacob et al., 2023; Kam et al., 2013; Park et al., 2021; Rutter et al., 2009; S. Tikka et al., 2020; Vignapiano et al., 2019; Won et al., 2018; Zeev-Wolf et al., 2018 (N=16) | Alamian et al., 2020; Chang et al., 2024; S. Hirano et al., 2018; Y. Hirano et al., 2015; S. Kim et al., 2020; Koshiyama et al., 2021, 2022; Tagawa et al., 2022; Tanaka-Koshiyama et al., 2020; Venables et al., 2009; Yeh et al., 2023 (N=11) | N=0 | N=0 | | |  |  |
| MEAN DURATION OF ILLNESS (months) | | | | | | |  | | |
| *<12* | *12-60* | *61-120* | *121-240* | *>240* | *Na* | | |  |  |
| Sun et al., 2020  (N=1) | Andreou et al., 2014a; Andreou et al., 2014b; Jonak et al., 2019; Kikuchi et al., 2011; Krukow et al., 2018, 2020; Lottman et al., 2019; Mitra et al., 2015; Takahashi et al., 2018; S. Tikka et al., 2020; Yadav et al., 2021  (N=11) | Chen et al., 2024; J. S. Kim et al., 2014; S. K. Tikka et al., 2015  (N=3) | Baradits et al., 2019; Di Lorenzo et al., 2015; Gordillo et al., 2023; S. Hirano et al., 2018; Y. Hirano et al., 2015; Ibáñez-Molina et al., 2023; S. Kim et al., 2020; Koshiyama et al., 2022; Vignapiano et al., 2019; Won et al., 2018; Yeh et al., 2023 (N=11) | Freche et al., 2020; Koshiyama et al., 2021  (N=2) | Alamian et al., 2020; Arikan et al., 2018; Chang et al., 2024; Garakh et al., 2015; Grent-’t-Jong et al., 2018; Hanslmayr et al., 2012; Jacob et al., 2023; Kam et al., 2013; Ramyead et al., 2016; Rutter et al., 2009; Tagawa et al., 2022; Umesh et al., 2018; Venables et al., 2009; Zeev-Wolf et al., 2018 (N=14) | | |  |  |
|  | **ADMISSION STATUS** | | | | |  | | |  |
| *Inpatient* | | | *Outpatient* | | | *Na* | | |  |
| Andreou et al., 2014a; Andreou et al., 2014b; Chang et al., 2024; Freche et al., 2020; Garakh et al., 2015; Gordillo et al., 2023; Hanslmayr et al., 2012; Krukow et al., 2018; Lee et al., 2020; Lottman et al., 2019; Zeev-Wolf et al., 2018  (N=11) | | | Andreou et al., 2014a; Andreou et al., 2014b; Di Lorenzo et al., 2015; Kam et al., 2013; Kikuchi et al., 2011; J. S. Kim et al., 2014; Umesh et al., 2018; Venables et al., 2009 ; Arikan et al., 2018; Gordillo et al., 2023; Jacob et al., 2023; Koshiyama et al., 2021, 2022; Krukow et al., 2020; Lee et al., 2020; Lottman et al., 2019; Soni et al., 2020; Takahashi et al., 2018; Vignapiano et al., 2019; Yeh et al., 2023; Zeev-Wolf et al., 2018; Sun et al., 2021 (N=21) | | | Alamian et al., 2020; Baradits et al., 2019; Chen et al., 2024; Grent-’t-Jong et al., 2018; S. Hirano et al., 2018; Y. Hirano et al., 2015; Ibáñez-Molina et al., 2023; Jonak et al., 2019; S. Kim et al., 2020; Mitra et al., 2015; Park et al., 2021; Ramyead et al., 2016; Rutter et al., 2009; Tagawa et al., 2022; Tanaka-Koshiyama et al., 2020; S. K. Tikka et al., 2015; Won et al., 2018 (N=17) | | |  |

^#^equivalent BPRS score; °only negative symptoms evaluated; FEP first episode of psychosis

**Supplementary table S4**: statistics

|  | test |
| --- | --- |
| *Rutter 2009* | - t-test |
| *Venables 2009* | - MANCOVA - Wilcoxon pairwise test - Pearson’s correlations |
| *Kikuchi 2011* | - Unpaired two-tailed t-test |
| *Hanslmayr 2012* | - Chi2 - Mann-Whitney |
| *Andreou 2014a* | - t-test - network-based statistics |
| *Andreou 2014b* | - ANOVA - non-parametric randomization - network-based statistics |
| *Garakh 2014* | - t-test - ANOVA |
| *Kam 2014* | - ANOVA |
| *Kim 2014* | - Independent t-test - permutation analysis |
| *Di Lorenzo 2015* | - Log-F ratio statistics - Bonferroni correction |
| *Hirano 2015* | - ANOVA - Greenhouse-Geisser correction |
| *Mitra 2015* | - Mann-Whithney U-test - ANOVA - Friedman’s test - Spearman’s correlation - Bonferroni correction |
| *Tikka 2015* | - ANOVA - MANOVA - Pearson’s chi2 |
| *Ramyaed 2016* | - t-test - statistical non-parametrical mapping - linear mixed-effects model using LPS values |
| *Won 2017* | - t-test - chi2 - ANCOVA |
| *Arikan 2018* | - Non-parametric correlation analysis |
| *Baradits 2018* | - Random regression hierarchical linear model |
| *Grent-t’-Jong 2018* | - Non-parametric Montecarlo permutation statistics |
| *Hirano 2018* | - ANOVA - non-parametri Spearman rho test |
| *Jonak 2018* | - ANCOVA - Pearson’s correlation |
| *Krukow 2018* | - ANCOVA - ANOVA - Tukey test for unequal groups |
| *Takahashi 2018* | - Independent and paired t-test - ANCOVA - Spearman’s rank order correlations |
| *Umesh 2018* | - ANOVA - Pearson’s correlation - linear discriminant functional analysis |
| *Zeev-Wolf 2018* | - ANOVA |
| *Lottman 2019* | - Pearson’s correlations |
| *Vignapiano 2019* | - TANOVA - TANCOVA |
| *Alamian 2020* | - Two-tailed, unpaired, pseudo t-test - Pearson’s correlations |
| *Freche 2020* | - Mass univariate analysis approach - Wilcoxon test |
| *Kim 2020* | - Chi2 - ANOVA - Partial Pearson’s correlation |
| *Krukow 2020* | - ANOVA - ANCOVA |
| *Lee 2020* | - Chi2 - permutation test |
| *Soni 2020* | - K-means method - nonparametric permutations - t-test |
| *Tanaka-Koshiyama 2020* | - Chi2 - t-test - ANCOVA |
| *Koshiyama 2021a* | - General linear model - independent t-test |
| *Koshiyama 2021b* | - Linear mixed modeling |
| *Sun 2021* | - Repeated measures ANOVA - Greenhouse-Geisser correction - Spearman correlation analysis |
| *Yadav 2021* | - MANOVA - ANOVA - Pearson’s correlation coefficient |
| *Gordillo 2022* | - ANCOVA - η^2^ calculation - Pearson’s, partial least square and distance correlations - Elastic net regression model |
| *Tagawa 2022* | - Mann-Whithney rank test - multiple stepwise linear regression |
| *Ibanez-Molina 2023* | - Wilcoxon signed rank test |
| *Jacob 2023* | - Unpaired t-test - Pearson‘s correlations |
| *Yeh 2023* | - Non-parametric permutation randomization |
| *Chang 2024* | - t-test - Wilcoxon rank sum test |
